# Low-dose anti-inflammatory combinatorial therapy reduced cancer stem cell formation in patient-derived preclinical models for tumour relapse prevention

**DOI:** 10.1038/s41416-018-0301-9

**Published:** 2019-02-04

**Authors:** Bee Luan Khoo, Gianluca Grenci, Joey Sze Yun Lim, Yan Ping Lim, July Fong, Wei Hseun Yeap, Su Bin Lim, Song Lin Chua, Siew Cheng Wong, Yoon-Sim Yap, Soo Chin Lee, Chwee Teck Lim, Jongyoon Han

**Affiliations:** 1 BioSystems and Micromechanics IRG, Singapore-MIT Alliance for Research and Technology Centre, Singapore, Singapore; 2 Mechanobiology Institute, National University of Singapore, Singapore, Singapore; 3 Department of Biomedical Engineering, National University of Singapore, Singapore, Singapore; 4 Department of Biochemistry, Yong Loo Lin School of Medicine, National University of Singapore, Singapore, Singapore; 5 Singapore Centre for Environmental Life Sciences Engineering, Singapore, Singapore; 6 Singapore Immunology Network, Agency for Science, Technology and Research, Singapore, Singapore; 7 NUS Graduate School for Integrative Sciences & Engineering (NGS), National University of Singapore, Singapore, Singapore; 8 Lee Kong Chian School of Medicine, Nanyang Technological University, Singapore, Singapore; 9 Department of Medical Oncology, National Cancer Centre Singapore, Singapore, Singapore; 10 Department of Hematology–Oncology, National University Cancer Institute, National University Hospital, Singapore, Singapore; 11 Cancer Science Institute of Singapore, National University of Singapore, Singapore, Singapore; 12 Biomedical Institute for Global Health Research and Technology, National University of Singapore, Singapore, Singapore; 13 Department of Electrical Engineering and Computer Science, Department of Biological Engineering, Massachusetts Institute of Technology, Cambridge, Massachusetts USA

## Abstract

**Background:**

Emergence of drug-resistant cancer phenotypes is a challenge for anti-cancer therapy. Cancer stem cells are identified as one of the ways by which chemoresistance develops.

**Method:**

We investigated the anti-inflammatory combinatorial treatment (DA) of doxorubicin and aspirin using a preclinical microfluidic model on cancer cell lines and patient-derived circulating tumour cell clusters. The model had been previously demonstrated to predict patient overall prognosis.

**Results:**

We demonstrated that low-dose aspirin with a sub-optimal dose of doxorubicin for 72 h could generate higher killing efficacy and enhanced apoptosis. Seven days of DA treatment significantly reduced the proportion of cancer stem cells and colony-forming ability. DA treatment delayed the inhibition of interleukin-6 secretion, which is mediated by both COX-dependent and independent pathways. The response of patients varied due to clinical heterogeneity, with 62.5% and 64.7% of samples demonstrating higher killing efficacy or reduction in cancer stem cell (CSC) proportions after DA treatment, respectively. These results highlight the importance of using patient-derived models for drug discovery.

**Conclusions:**

This preclinical proof of concept seeks to reduce the onset of CSCs generated post treatment by stressful stimuli. Our study will promote a better understanding of anti-inflammatory treatments for cancer and reduce the risk of relapse in patients.

## Introduction

In the recent decade, there has been an increasing number of anti-cancer drug clinical trials.^1^ However, the efficacy of several drugs may be limited by the requirement for higher dosage in vivo to overcome pharmacokinetics issues.^2^ Another key factor in the lack of therapeutic efficacy is the inability to eliminate cancer cells completely, a process hindered by the heterogeneity and plasticity of human biological systems.^3,4^ Notably, stressful stimuli post treatment are known to have either a prodeath or prosurvival role and could drive cancer cells to become more metastatic and drug-resistant.^5^

The reduction of cancer stem cells (CSCs) post treatment is important as the emergence of CSCs via epithelial–mesenchymal transition (EMT) is identified as one of the ways by which chemoresistance develops.^6–8^ Other ways involve transporter pumps,^9^ genetic alteration,^10^ or exosomes.^11^ Hence, CSCs as key targets for anti-cancer strategies.^12^ CSCs may be found circulating in the bloodstream^13^ upon extrusion by primary tumours.^14^ Heterogeneity and plasticity of CSCs hinder complete eradication,^15^ which account for metastasis^16^ at distant sites even after successful treatment.^17^

It was previously shown that cancer patients on a supplement of aspirin had reduced cancer risk and longer overall survival than those who were not.^18,19^ Aspirin is a nonsteroidal anti-inflammatory drug most commonly used to treat inflammatory diseases. The association between chronic inflammation and cancer^20,21^ suggests that aspirin can be effective against cancer. Indeed, anti-cancer effects of aspirin have been established in colorectal cancer,^19,22,23^ oesophageal cancer,^24^ gastric cancer,^25^ liver cancer,^26^ and pancreatic cancer.^27^

In this proof of concept study, a range of therapeutic drug concentrations for 0–500 mg/ml aspirin (A) and 0–1 µM doxorubicin (D), a common anti-cancer drug for breast cancer, were screened with a microfluidic culture and drug-screening assay validated for primary cell cultures.^28^ We demonstrated that low doses of aspirin ( ≤ 500 mg/ml) in combination with sub-optimal doses of doxorubicin, a chemotherapy drug, could heighten anti-cancer effect within a relatively short period of time (72 h), specifically in breast cancer cell lines and patient-derived clinical models. Cells treated with doxorubicin alone demonstrated an increase in CSC proportion over time (7 days). Conversely, cells under combinatorial DA treatment generated a significantly lower proportion of CSCs, leading to reduced cancer cell cluster formation or spheroid growth.

Under combinatorial DA treatment, there was also a reduction of metastatic-like phenotype as compared with cells treated with doxorubicin alone. This was despite the increase of interleukin-6 (IL-6) and *JAK1* expression levels, which was owing to the inhibition of IL-6 by combinatorial DA treatment, leading to an overall reduction of CSCs.^29,30^ Combinatorial treatment also reduced oxidative stress in the cells, as evident by Calcein AM expression, 3-(4,5-Dimethyl-2-thiazolyl)-2,5-diphenyl-2H-tetrazolium bromide (MTT) and peroxidase assays. The effects of combinatorial DA treatment were also mediated by cyclooxygenase (COX)-related pathways. Prior studies have demonstrated that COX-2/prostaglandin E2 (PGE2) pathways are potent inhibitors of EMT for epithelial cells,^31^ and the resultant COX-2-derived PGE2 and PGD2 are mediators of anti-EMT.^32^ COX-2 was also highly expressed in triple-negative breast cancer and is associated with poorer prognosis.^33^

We demonstrated that the reduction of CSCs under combinatorial DA treatment was reflected in both the cancer cell clusters and patient-derived circulating tumour cells (CTC) cluster models. The CTC clusters were obtained under culture with our microfluidics assay with minimal processing, which vastly promotes efficiency and allows samples to be analysed after 2 weeks.^34^ Specialised microwells within the assay recapitulate the tumour microenvironment through the proximity of cancer cells, and co-culture with patient’s own immune cells under hypoxia. Doxorubicin has been reported in several cases as ineffective in the eradication of resistant CSCs.^35,36^ To the best of our knowledge, our study is the first to connect the anti-cancer effects of aspirin and anti-cancer drugs with the reduction of CSCs, in a patient-specific manner.

Our results suggest that the combinatorial use of aspirin and doxorubicin have the potential to prolong overall patient survival, paving the way for further studies on the co-treatment of other chemotherapy drugs with aspirin. Such studies also demonstrate the potential to reinvent a new role for aspirin, an affordable drug that can be accessed by many patients globally. Although the efficacy of combinatorial drug regime would be heterogeneous among patient populations, CTC cluster-forming assay would allow one to quickly evaluate this for optimal management of metastasis. These preclinical studies will potentially benefit patients by reducing the risk of cancer relapse after treatment and revolutionise personalised medicine through cheap and non-invasive screening methods.

## Results

### Preclinical drug-screening using the high-throughput CTC cluster assay

**Fig. 1:**
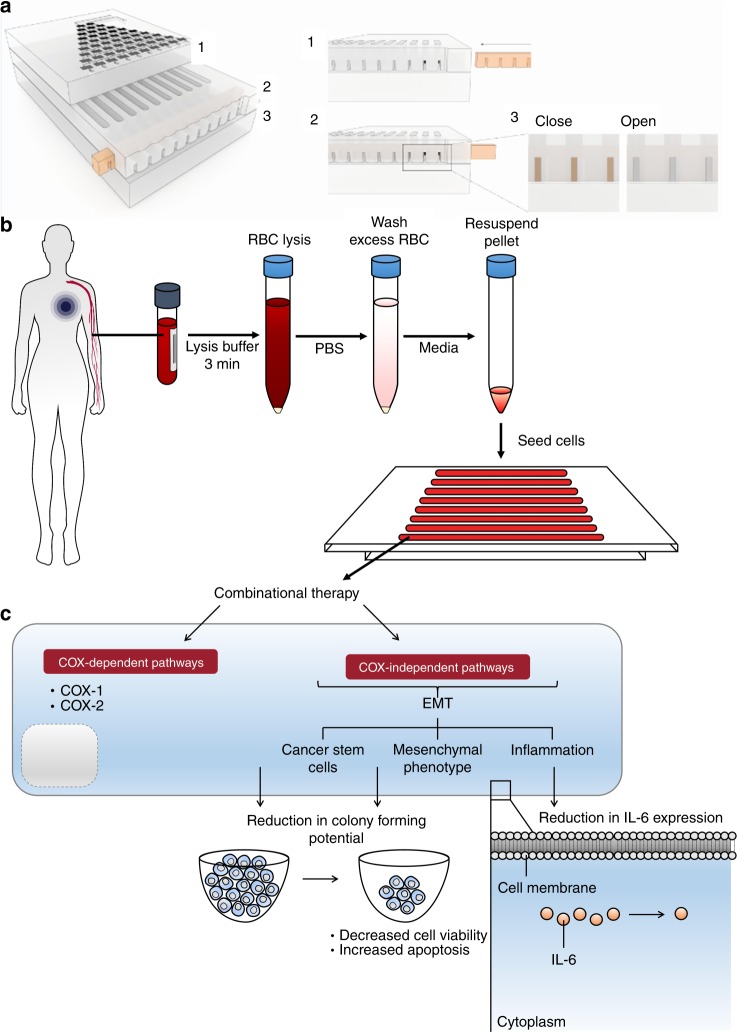
Overview of assay design, screening, and therapeutic outcomes. **a** Schematics of the complete device (left panel), with details of valve design. In this portion of the device, the open channels were operated with an open/closed valve actuated by simply sliding in or out a bar with openings corresponding to the position of the channels. **b** Workflow for cancer cell cluster establishment and subsequent downstream analyses. A tumour-on-chip-like assay can be established under factors that aim to mimic the tumour microenvironmental niche, using a single pre-processing step to remove red blood cells prior to seeding. **c** The proposed mechanism of anti-inflammatory combinatorial treatment screened with the preclinical patient-derived model

We first utilised a cancer cell cluster assay optimised for cluster formation of clinical primary CTCs from blood biopsy^37^ as a relevant model in vitro for uniform cluster formation. The assay was designed to incorporate a layer of customised ellipsoidal microwells of 250 µm by 150 µm and 150 µm depth, to promote the formation of single cancer cell clusters at low cell seeding concentration (0.25 million cells per channel). The assay was integrated with microfluidic components to enable high-throughput screening of drug combinations, as well as valves to control and compartmentalise media waste disposal (Fig. 1a, Figure S1A, B). The design of the assay is flexible owing to the use of microfabrication, and assays can be incorporated into any number of channels, which corresponds to the range of drug concentrations screened. In this work, we utilised either the 8-channel or 10-channel device for drug-screening tests where the drugs administered can vary up to 10 different concentrations (*n* = 8 or 10) (Figure S1C) either with one or two of the drug concentrations (Figure S1D, E) by pump infusion or manual addition (Figure S1F).

The cancer cell cluster formation process involved the seeding of cells into the microwells, followed by maintenance under a combination of conditions such as hypoxia to mimic the tumour microenvironmental niche (Fig. 1b). Resultant cancer cell clusters were three-dimensional, multilayered, and under low shear.^37^ Minimal pre-processing reduced the damage to target CTCs and allow increased efficiency of CTC cluster formation.^28,34,38^ Resultant clusters were heterogeneous and contain subpopulations of cancer stem cells, depending on sample parameters. The presence of CTCs, CSCs and other constituents was previously identified by immunostaining and/or fluorescence in situ hybridisation.^38^ Other constituents identified within the clusters include residual immune cells, some of which were also populations located in the tumour microenvironment niche. Despite the label-free and growth factor-free conditions, there was still an extent of selection in our assay with respect to the proliferative and cluster-forming CTC populations. Nonetheless, these populations are of utmost relevance to cancer progression, and hence reflect the potential of patient’s response to therapy.

Consistent cancer cell clusters by size could be generated within the microwells (Figure S2A) with various cancer types such as colorectal and breast cancer cell lines. The range of cluster diameter for a single sample was consistent throughout the assay (SW480: 136.1 ± 7.1 µm, MDA-MB-231: 142.2 ± 4.6 µm; Figure S2B) but comparison of cluster sizes across cell lines of different EMT phenotypes demonstrated that epithelial-like cancer cell lines led to smaller cancer cell clusters, possibly due to closer cell–cell interactions (Figure S2C).^39^

To evaluate the drug response after combinatorial DA therapy, we first validated the cellular viability of monolayer cultures after respective drug exposures. For cellular viability, we validated the IC50 values of aspirin alone, doxorubicin alone or combinatorial DA therapy on MDA-MB-231 as 2D monolayers (seeding density 10k cells; Figure S3). We confirmed that the range of aspirin concentrations used was mostly above median lethal dosage (IC50 value = 412.5 mg/ml), whereas concentrations for treatment with doxorubicin alone (IC50 value = 0.1 µM) and combinatorial DA treatment with doxorubicin concentration fixed at 0.5 µM (0.5 D) were within 50% lethal dosage range. In fact, viability rates of monolayer cultures after combinatorial DA therapy were below that of cultures after single 0.5 D treatment, suggesting a higher killing efficacy. Viability rates were determined by live and dead staining using Calcein AM and SYTOX, respectively. Using these optimal drug concentrations, we evaluated killing efficacy, metastatic potential and the mechanisms of treatment using our preclinical model on cancer cell lines and clinical patients (Fig. 1c).

### Combinatorial anti-inflammatory therapy improved killing efficacy and apoptosis onset

**Fig. 2:**
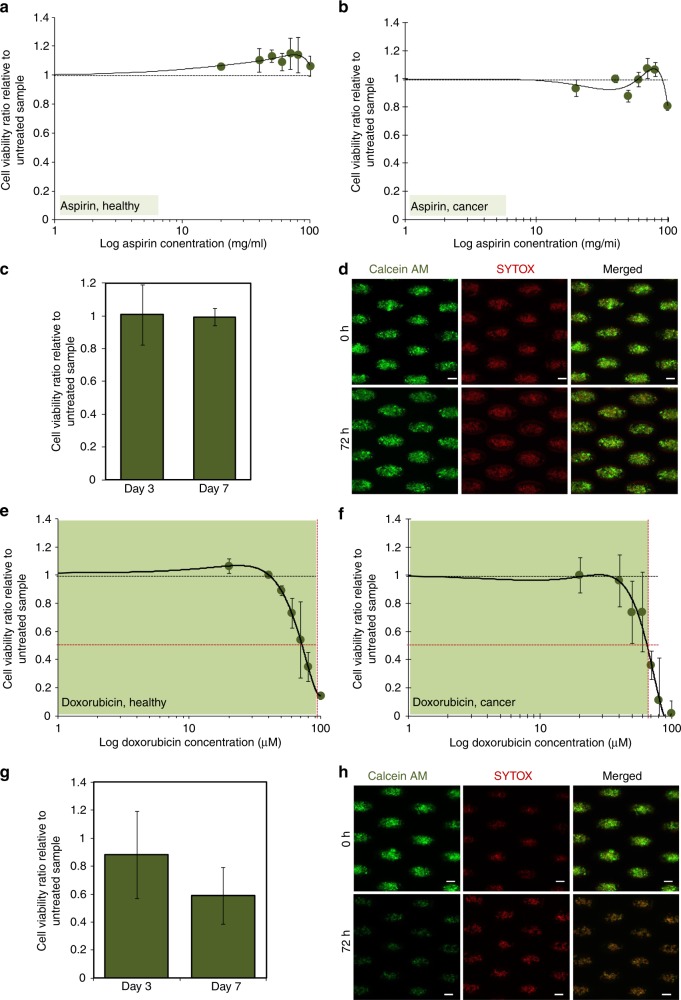
Assay outcome for single drug exposure using aspirin or doxorubicin. Viability ratios of **a** healthy and **b** MDA-MB-231 cancer cell cultures after 72 h drug exposure under the different percentage of aspirin concentrations (0–200 mg/ml), relative to averaged viability percentage of untreated cultures (i.e., 0 mg/ml aspirin concentration). Blood cells and cell lines serve as controls for components of the clinical patient samples, mainly white blood cells and cancer cells, respectively. Black dotted reference line indicates the averaged viability of the vehicle groups.(**c** Viability rates remained relatively constant after 72 h or 7 days of exposures to 250 mg/ml aspirin. **d** Representative images of live/dead staining of untreated cluster cultures (top) and cultures after 72 h treatment (bottom). Scale bar is 100 µm. The viability ratio of **e** healthy and **f** MDA-MB-231 cancer cell cultures after 72 h drug exposure with different percentage of doxorubicin concentrations alone, relative to averaged viability percentage of untreated cultures (i.e., 0 µM doxorubicin concentrations). The inhibitory concentration at 50% viability (IC50) values (horizontal dotted line) was obtained based on the corresponding drug concentration value that reflected 50% cell viability as indicated on the graphs by the vertical dotted lines (healthy cultures: 0.84 µM; cancer cell culture: 0.75 µM). Green regions show the concentrations selected for evaluation of long-term cultures for subsequent drug experiments. Black dotted reference line indicates the averaged viability of the vehicle groups. **g** Viability decreases after prolonged exposure to 0.5 µM doxorubicin. *p* = 0.189, with respect to differences in cell viability for cultures treated with doxorubicin only for 7 days and 72 h. **h** Representative images of live/dead staining of untreated cluster cultures (top) and cultures after 72 h treatment (bottom). Scale bar is 100 µm

We next tested the combinatorial DA therapy of doxorubicin and aspirin (DA) on three-dimensional (3D) cancer cell clusters. When cancer cell clusters were untreated over 7 days, viability rates of these cultures remained relatively constant (*p* < 0.01; Figure S4). Vehicle samples were subjected to the same dimethyl sulfoxide (DMSO) concentration as those under respective drug treatments. After treatment with aspirin alone, viability rate of healthy blood cells remained constant after short-term exposure (72 h) to varied dosages of aspirin (0–500 mg/ ml) (Fig. 2a, b). After treatment with only 250 mg/ml aspirin concentration, the viability of the cultures remained constant over exposure periods of both 72 h and 7 days (*p* < 0.01), relative to untreated samples (Fig. 2c, d) (Figure S5A). After treatment with doxorubicin alone, viability percentage dropped sharply beyond drug concentrations of 0.65 µM (65%) for both cultures of healthy blood cells (Inhibitory concentration at 50% viability (IC50) = 0.75 µM) and cancer cell line (IC50 = 0.84 µM)) (Fig. 2e, f) (Figure S5B).

To investigate if the addition of aspirin would increase the anti-cancer effects of doxorubicin, we only used drug concentration levels that will yield minimal toxicity ( > 50% viability after 72 h treatment), hence only 0–50% (0–0.5 µM) of doxorubicin concentrations were used for subsequent combinatorial therapy experiments (Figure S6). For the treatment with 0.5 µM doxorubicin concentration alone (0.5 D), viability ratio could be reduced to 0.59 ± 0.2 after 7 days of exposure, relative to untreated samples (Fig. 2g, h), although the reduction was not significant.

**Fig. 3:**
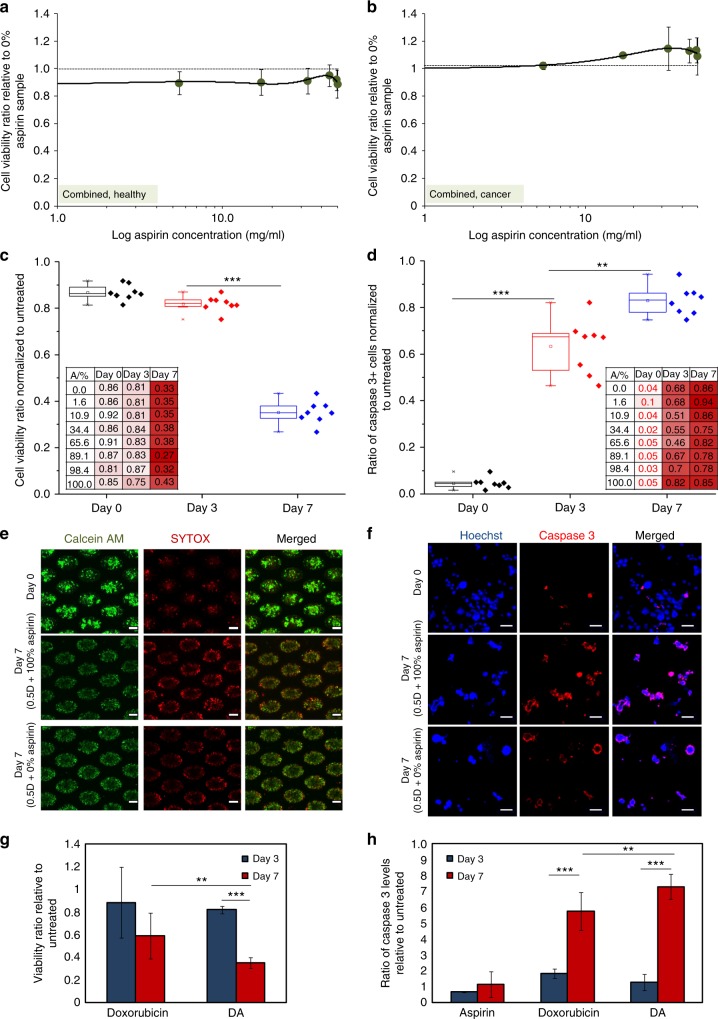
Viability rates under Combinatorial DA treatment administration. The viability ratio of **a** healthy and **b** MDA-MB-231 cultures after 72 h drug exposure to combinatorial DA treatment with different concentrations of aspirin only and fixed concentrations of doxorubicin at 0.5 µM (0.5 D), relative to the averaged viability percentage of cultures treated only with 0.5 D. Black dotted reference line indicates the averaged viability of the vehicle groups. **c** Viability ratio decreases after prolonged exposure to combinatorial DA treatment. Viability proportion varied with aspirin concentration under combinatorial therapy (inset). ***p < 0.00001. **d** The proportion of apoptotic cells (caspase-3 positive) increases after prolonged exposure to combinatorial DA treatment. Viability proportion varied with aspirin concentration under combinatorial therapy (inset). ***p < 0.00001, **p < 0.01. **e** Representative images of cancer cell clusters stained with live/dead dyes of untreated cluster cultures (top) and cultures after 72 h treatment (bottom). Scale bar is 100 µm. **f** Representative images of resuspended single cells from cluster cultures, stained with Caspase-3 antibody and nuclei dye. Scale bar is 20 µm. Killing efficacies of drug treatment with doxorubicin alone and combinatorial DA treatment via **g** comparison of viable cell proportions and **h** comparison of Caspase-3^+^ cell proportions. ***p < 0.00001, **p < 0.01

Viability ratios of cultures with respect to samples treated only with 0.5 D were obtained to determine the effects of combinatorial treatment with aspirin. Vehicle samples were subjected to the same DMSO concentration as those under respective drug treatments. Under combinatorial DA treatment, viability proportions remained relatively constant after short-term exposure (72 h) to different concentrations of aspirin in combination with 0.5 D for both healthy and cancer cell cultures (Fig. 3a, b). However, after 7 days of exposure with combinatorial DA treatment, viability ratio, normalised to that of untreated, was significantly reduced to an average value of 0.35 ± 0.05 (*p* < 0.00001; Fig. 3c). The highest killing efficacy on Day 7 was observed at 482.5 mg/ml aspirin concentration in combination with 0.5 D (*p* = 0.039). Intriguingly, the ratio of apoptotic cells, normalised to that of untreated, after combinatorial DA treatment increased significantly even within 72 h of exposure (63 ± 0.1%, *p* < 0.00001) and was maximised after 7 days of exposure (83 ± 0.06%, *p* = 0.0009; Fig. 3d), as compared with treatment with doxorubicin alone. The highest proportion of apoptotic cells was also obtained under treatment within 445–500 mg/ml aspirin concentration in combination with 0.5 D. Hence, we demonstrated that the killing efficacy of combinatorial DA therapy, especially with 0.5 D and 445–500 mg/ml aspirin could be more effective than the killing efficacy administered by treatment with 0.5 µM doxorubicin alone (0.5 D) after 7 days of exposure (Fig. 3e–h) (Figure S7A). Killing efficacy of DA treatment significantly surpassed that of treatment with doxorubicin alone after 7 days of exposure, as determined by the live and apoptotic cell proportions (*p* < 0.01; Fig. 3g, h) (Figure S7B).

### Combinatorial anti-inflammatory therapy reduced gene and protein expressions associated with the formation of cancer stem cells

**Fig. 4:**
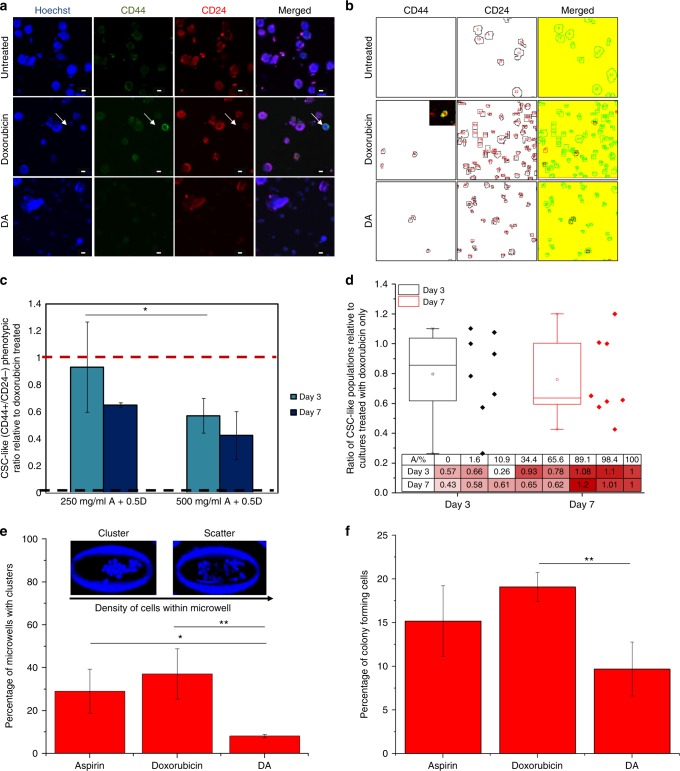
Combinatorial DA treatment reduced the proportion of cancer stem cells. **a** Representative images of cancer cell clusters stained with CD44 and CD24. Scale bar is 20 µm. Doxorubicin concentration was 0.5 µM and combinatorial DA treatment was 500 mg/ml aspirin with 0.5 µM. **b** Images of CD44/CD24 stained cells after processing with image analysis software for quantification of CD44^+^/CD24^−^ cell proportions. Doxorubicin concentration was 0.5 µM and combinatorial DA treatment was 500 mg/ml aspirin with 0.5 µM. **c** Relative proportion of CD44^+^/CD24^−^ cells under DA treatment with low-dose (~ 327.5 mg/ml) or high-dose (~ 500 mg/ml) aspirin concentrations generated in the assay. Black and red dash lines indicate average proportions of CSC in untreated and cells treated only with doxorubicin respectively. *p < 0.05 (*p* = 0.022). **d** Box plot demonstrating the suppression of CSC-like phenotype (CD44^+^/CD24^-^) with respect to doxorubicin exposure alone. (**e**) The proportion of microwells with cancer cell clusters after treatment was the lowest under combinatorial DA treatment. Cluster phenotype was determined by the density of cells per unit area.^72^ *p < 0.05 (*p* = 0.037), ** *p* < 0.01. **f** Plot demonstrating reduced colony formation ability in agarose gels when cultures were exposed to combinatorial treatment relative to single drug administration. (Top) Representative images of a stained cancer cell cluster (Calcein AM/Hochest) in agarose gel. ** *p* < 0.01

It has been reported that secondary tumours in cases of relapse after treatment are often multidrug-resistant.^40^ This observation may be attributed to an incomplete eradication of heterogeneous cell populations under targeted anti-cancer therapeutic strategies.^41^ Here, we support this observation by demonstrating an increase in proportion in CD44^+^/CD24^−^ phenotype cells after exposure of cancer cell clusters to doxorubicin. Prior studies have also demonstrated that an increase of CD44^+^/CD24^−^ phenotypes in epithelial-like luminal breast cancer subtypes lead to a heightened resistance to chemotherapy.^42^ The CD44^+^/CD24^−^ phenotype corresponded to the proportion of CSC-like cells,^43^ and they were identified by immunostaining of the harvested cluster cultures after 7 days of exposure to 0.5 µM of doxorubicin (Fig. 4a, b) (Figure S8). Direct cohort measurements were obtained instead of conventional methods such as fluorescence-assisted cell Sorting, to prevent loss of target cells in these heterogeneous populations. Increase in CSCs post treatment is undesirable as they have a high potential to form secondary metastases, leading to cancer relapse.^44^

Setting the averaged ratio of CD44^+^/CD24^−^ CSCs generated upon treatment with 0.5 µM of doxorubicin alone as 1 and untreated samples as 0.058 ± 0.008, we assessed the proportion of CSCs in combinatorial DA-treated samples **(**Fig. 4c). CSC pool size varied with treatment condition, ranging from 5.5 to 26.3% in treated cohorts. Untreated cohorts had a CSC pool size of below 1.2 ± 0.02%. Combinatorial DA treatment with 250 mg/ml aspirin reduced the proportion of CSCs slightly (ratio: 0.93 ± 0.33, 72 h) but at combinatorial DA treatment with 500 mg/ml aspirin resulted in a significant reduction of CSC proportion to a ratio of 0.57 ± 0.13 (72 h). The reduction in CSC levels of cultures was much significant than the overall reduction in cellular viability (Fig. 3c) over 72 h or 7 days treatment, hence the reduction of CSC cohorts under combinatorial DA treatment was not a result of the selective killing of non-CSCs (Figure S8). The overall CD44 expression was also significantly lower under combinatorial DA treatment as compared with single doxorubicin treatment (Figure S9). Hence, under combinatorial DA therapy, the averaged proportion of CSC-like phenotype was reduced over time with increasing doses of aspirin (Fig. 4d). Despite the overall change in CD44^+^/CD24^−^ phenotype proportions, expression levels of CD44 in CD44^+^cells did not seem to vary significantly (Figure S10).

When treated cells were transferred to a new microwell-based cluster assay to determine cluster-forming potential,^34^ combinatorial DA treatment (250 mg/ml A + 0.5D) led to a significant reduction (0.22:1 ratio) in cancer cell cluster-forming capability as compared with treatment with doxorubicin alone (*p* < 0.01, Fig. 4e). The known reported percentage of colony-forming cells in untreated MDA-MB-231 is < 5% (0.05 ratio).^45,46^ Intriguingly, treatment with aspirin alone was not expected to influence cluster formation, yet a higher cancer cell cluster formation potential (3.57:1 ratio) was observed for single aspirin treatment (0.5 A) as compared with combinatorial DA treatment (*p* = 0.037). A similar trend was observed when treated cells were transferred to three-dimensional agarose gels for spheroid formation, and the proportion of cells forming spheroids was significantly lowest in combinatorial DA-treated cells and highest in cells treated with doxorubicin only (*p* < 0.01, Fig. 4f, Figure S11).

### Heterogeneous response of therapy in clinical cohorts highlights the importance of patient-derived preclinical models

**Fig. 5:**
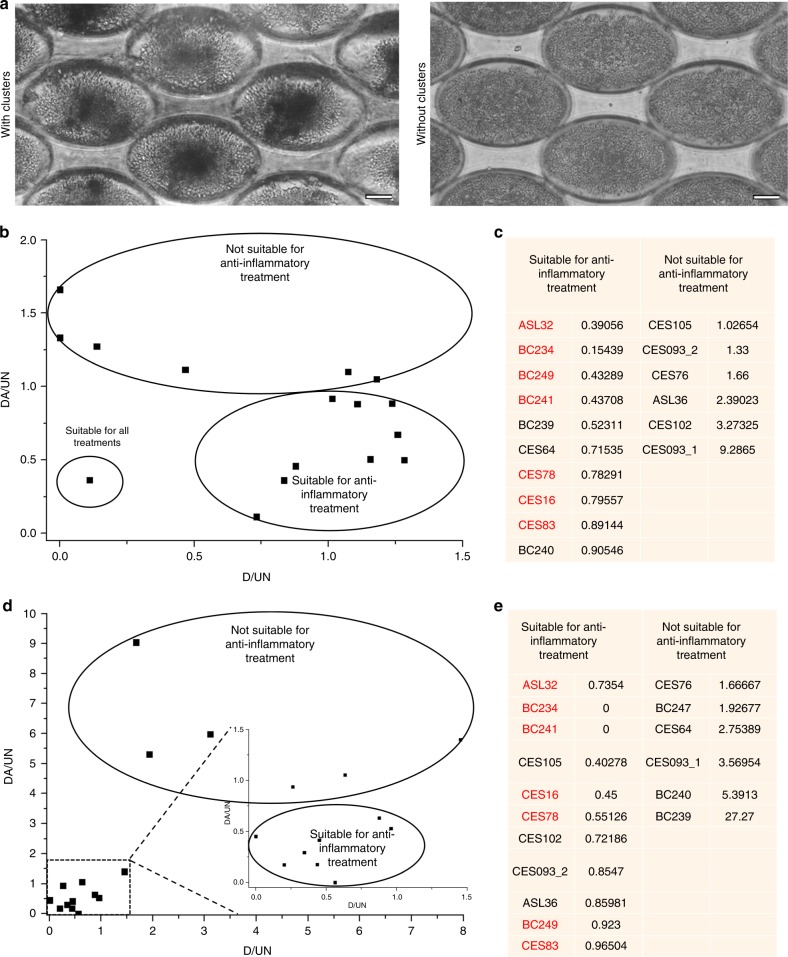
Evaluation of Combinatorial DA treatment on patient-derived CTC models using the Cluster assay. **a** Representative images of microwell array with clusters (left) and without clusters (right). Scale bar is 50 µm. **b** Scatter plot demonstrating the killing efficacy in clinical cohorts. DA/UN = viability of putative CTCs in patient-derived models after 72 h of treatment under combinatorial DA treatment, relative to untreated samples. D/UN = viability of putative CTCs in patient-derived models after 72 h of treatment under treatment with doxorubicin alone, relative to untreated samples. The response in terms of killing efficacy is heterogeneous with samples of comparable response grouped in black. **c** List of patients suitable or unsuitable for anti-inflammatory combinatorial DA treatment based on viability ratio. Patient samples that responded in terms of both killing efficacy and CSC reduction are marked in red. **d** Scatter plot demonstrating the reduction of CSCs in clinical cohorts. DA/UN = CSC counts of putative CTCs in patient-derived models after 72 h of treatment under combinatorial DA treatment, relative to untreated samples. D/UN = CSC counts of putative CTCs in patient-derived models after 72 h of treatment under treatment with doxorubicin alone, relative to untreated samples. The response in terms of reduction of CSCs is heterogeneous with samples of comparable response grouped in black. **e** List of patients suitable or unsuitable for anti-inflammatory combinatorial DA treatment based on CSC counts. Patient samples that responded in terms of both killing efficacy and CSC reduction are marked in red

We processed 68 clinical blood samples from patients with breast cancer using the preclinical CTC Cluster Assay to obtain positive cultures for combinatorial drug treatment evaluation. The assay demonstrated high efficiency of cluster formation, of which 60.9% of the samples demonstrated cluster positivity. This varied with treatment time point, with 72.0% positivity in baseline samples but 54.9% positivity in post treatment samples (Table S1). Of the 60.9% of positive samples, 17 samples were evaluated due to a minimum cell count per sample required for seeding of multiple channels to allow parallel evaluation of different treatment conditions (Table S2). Killing efficacy was more variable in patient-derived CTC clusters, reflecting the heterogeneity of clinical samples. In all, 62.5% of clinical samples demonstrated a higher killing efficacy with combinatorial DA treatment as compared with doxorubicin alone after 72 h exposure. CTCs in some clinical models were also eradicated more efficiently with doxorubicin alone (ASL36, CES93 and CES76), reflecting the heterogeneity of patient profiles and the importance for reliable patient-derived models to screen patients suitable for similar anti-inflammatory and anti-cancer strategies (Fig. 5a, b).

The reduction of CSCs in patient-derived CTC clusters was similarly varied across the cohort owing to the heterogeneity of clinical samples. 64.7% of clinical samples demonstrated a higher reduction in CSC proportions upon exposure to combinatorial DA treatment, as compared with doxorubicin alone. Some clinical samples demonstrated a reduction in CSCs even with doxorubicin alone but were further reduced under combinatorial DA treatment (38.9%, 7/18) (Fig. 5c). Some samples also reflected a significantly better response under treatment with doxorubicin only but not with combinatorial DA treatment (CES76 and BC239). One clinical sample (BC249), which was susceptible to both forms of treatment (*n* = 3) had corresponding tumour analysis demonstrating oestrogen positivity (ER+) (Table S2), and patients with ER+ subtype were widely reported to have a better overall survival.^47^ Overall, 41.2% of clinical samples reflected heightened treatment efficacy under combinatorial treatment only in terms of both killing efficacy and CSC reduction, reflecting the cohort that was most suitable for anti-inflammatory treatments.

### Combinatorial DA treatment revert cells to a less metastatic phenotype via suppression of signalling pathways involved in the EMT

An inhibition of COX enzymes is often attributed to the mechanism of aspirin.^48^ In non-neoplastic studies, low doses of aspirin could inhibit COX-1 for antithrombotic effects, and higher doses (> 650 mg per day) led to inhibition of both COX-1 and COX-2 for analgesic and antipyretic effects.^49^

COX enzymes are often unregulated in many cancers and are associated with inflammation.^50^ However, the exact mechanisms of these proposed anti-cancer properties of aspirin are much less understood. Prior reports have suggested that the inhibition of COX-2 may be key for its anti-cancer therapeutic effects.^51^ Yet, the observations remain inconclusive as alternative mechanisms independent of COX enzymes have also been suggested for the anti-cancer effects of aspirin.^52,53^

**Fig. 6:**
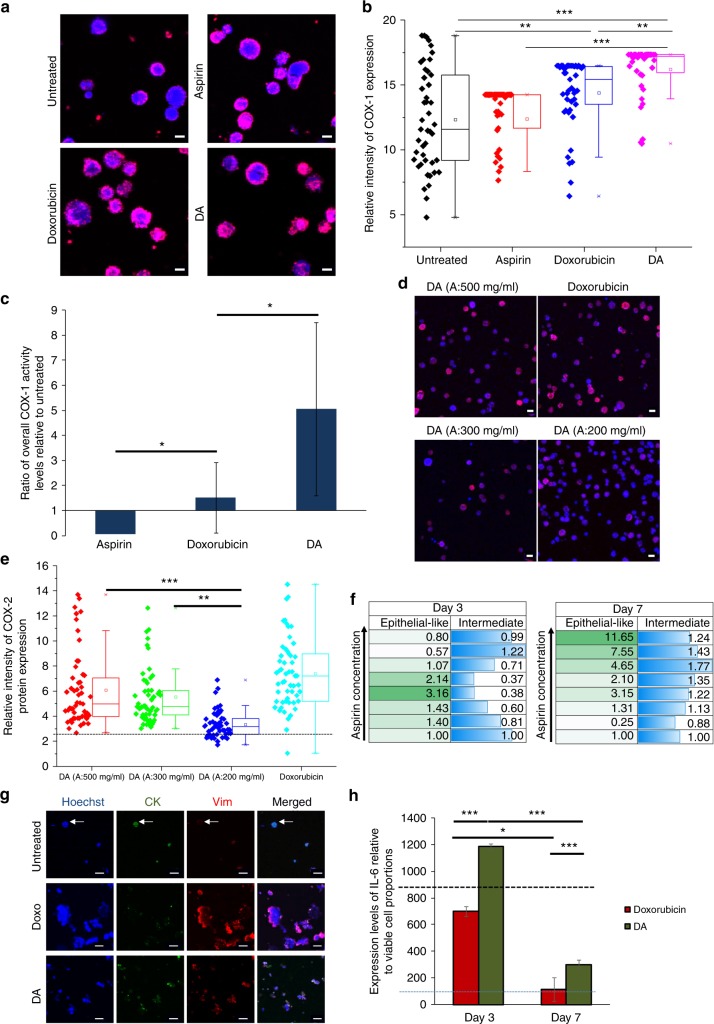
DA Combinatorial DA treatment acts via a COX-independent pathway and was mediated by anti-inflammatory mechanisms suppressing metastatic phenotypes. **a** Immunostaining for COX-1 protein on harvested cell cluster cultures. Scale bar is 10 µm. **b** Expression levels of COX-1 protein in cultured cells after 72 h of drug exposure normalised to background levels. ***p < 0.00001, **p < 0.01. **c** Relative levels of COX-1 activity levels of cultured cells after 72 h of drug exposure, as determined by the COX-1 activity assay. Aspirin and doxorubicin: *p < 0.05 (*p* = 0.043). Aspirin and combinatorial DA: *p < 0.05 (*p* = 0.012). **d** Immunostaining for COX-2 protein. DA refers to combinatorial treatment with 0.5 D and 500 mg/ml aspirin as indicated. Doxorubicin treatment refers to treatment with 0.5 µM dosage of doxorubicin. Scale bar is 10 µm. **e** Relative intensity levels of COX-2 protein after 7 days of drug exposure. ***p < 0.00001, **p < 0.01. **f** Proportions of epithelial-like (CK^+^Vim^−^) and intermediate EMT (CK^+^Vim^+^) phenotypes after combinatorial DA treatment, after 72 h or 7 days of drug exposure. Data are relative to that of cells treated only with doxorubicin. **g** Immunostaining for CK and Vimentin on harvested cell cultures. Arrows indicate an epithelial-like cell. Scale bar is 20 µm. **h** Cytokine IL-6 levels secreted under treatment with doxorubicin only and combinatorial DA-treated cells under exposure periods of 72 h and 7 days, as determined by ELISA assays. The expression levels are normalised to the viable cell proportions of each culture condition. Reference lines were provided for the average expression level of IL-6 in untreated cluster cultures after 72 h (black dotted line) and at 7 days treatment (the blue dotted line) respectively. *p < 0.05, ***p < 0.00001

We found that COX-1 protein expression was higher in both doxorubicin-treated and DA-treated cells, relative to untreated or aspirin-treated cells as determined by immunostaining (*p* < 0.01, Fig. 6a, b). There was also a significantly higher expression of the COX-1 protein in doxorubicin-treated cells as compared with untreated cells (*p* < 0.01, Fig. 6b). Similar to protein expression, COX-1 enzyme activity levels were also higher in combinatorial DA-treated cells as compared with cells treated with doxorubicin only (Fig. 6c), as demonstrated by the COX-1 activity assay. Interestingly, COX-2 protein expression was reduced under combinatorial DA treatment, as evident by immunostaining (Fig. 6d). This is important as the reduction of COX-2 is directly associated with malignancy and resistance.^54^ Reduction in COX-2 protein expression was significantly greater under low-dose aspirin (200 mg/ml), as compared with higher dosages (300 mg/ml aspirin (*p* < 0.01) and 500 mg/ml aspirin (*p* < 0.00001)) of aspirin in combinatorial treatments (Fig. 6e). These supported the notion that combinatorial DA treatment is more effective in its anti-cancer effects.

Cancer cells usually present as a hybrid phenotype and express intermediate levels of epithelial (E) and mesenchymal (M) characteristics. In this consideration, cancer cells are highly dynamic and can harvest benefits of both E (e.g., increased adhesion) and M phenotypes (e.g., stemness, resistance) to different degrees. Here, we found that there were also changes in their proportions of epithelial phenotypes after treatment with both doxorubicin alone and combinatorial treatments, as evident by the immunostaining for markers such as cytokeratin (CK) and Vimentin (Vim) on harvested cells or cell clusters in situ (Fig. 6f). CK is a cytoplasmic marker for epithelial phenotypes while Vim is a cytoplasmic marker for mesenchymal phenotypes.^55^ Specifically, the proportion of epithelial-like (CK^+^Vim^−^) cells was reduced after doxorubicin, which was most evident after 7 days of treatment. This observation was reversed under combinatorial DA treatment, with the increase of epithelial-like (CK^+^Vim^−^) cell populations. Under combinatorial DA treatment using a range of 7.8–328.1 mg/ml aspirin, the proportion of cells with epithelial-like phenotypes were increased within 72 h. This was more apparent after 7 days in treatment, with a notable increase of epithelial-like phenotypes in cells treated with combinatorial DA treatment using > 171.5 mg/ml of aspirin, along with a relative increase in intermediate EMT (CK^+^Vim^+^) subtypes (Fig. 6f, g) (Figure S12). Clusters also demonstrated the highest packing density in untreated cultures and were lowest in doxorubicin only treated cultures (Figure S13). The overall shift toward a more E phenotype is important as it suggests a reduction in stemness and drug resistance capabilities, an observation that was supported as well with the cluster and spheroid-forming assays (Fig. 4e, f).

The IL-6 inflammatory loop has also been associated with anti-cancer drug resistance through the expansion of CSCs.^56,57^ We demonstrated that the relative IL-6 expression as determined by ELISA normalised to the proportion of viable cells was reduced after 72 h treatment with doxorubicin only, but not combinatorial DA treatment (*p* < 0.00001, Fig. 6h). Interestingly, untreated cancer cells maintained as clusters over 7 days also demonstrated a reduction in IL-6 expression (~ 9.6 fold at Day 7 as compared with 72 h). The increase in IL-6 was matched by the corresponding increase in JAK1 gene expression, albeit we did not detect STAT3 expression in cultures under all conditions (Figure S14). Despite the higher IL-6 and JAK1 gene levels, the cytokines were not able to induce similar expansion of CSCs owing to inhibition of IL-6 under combinatorial treatment. Such inhibition of IL-6 under low-dose aspirin has been previously reported in both healthy and cancerous tissues.^29,30^ The inhibition of IL-6 receptors while maintaining IL-6 levels is important as IL-6 is reported to demonstrate anti-inflammatory properties and play important roles in metabolism.^58,59^ Disruption of the IL-6 gene results in increased systemic inflammation and reduced glucose tolerance.^60,61^ Hence, a delay of IL-6 inhibition under proposed combinatorial DA treatment could actually be beneficial and reduce ‘stressful’ stimuli post treatment that drives cancer cells to become more metastatic and drug-resistant.^5,62,63^

As Calcein AM is also an indicator of intracellular oxidative activity,^64^ we compared the relative Calcein AM staining intensity between untreated samples and samples treated only with aspirin, as well as combinatorial DA-treated samples with samples treated only with doxorubicin. Calcein AM intensity was higher in untreated samples (both cluster and 2D cultures) as compared with samples treated with aspirin only. Similarly, Calcein AM intensity was higher in samples treated with doxorubicin only (both cluster and 2D cultures) as compared with samples under combinatorial DA treatment (Figure S15). The differential staining intensity of Calcein AM after 7 days of treatment suggests a reduction in intracellular oxidative activity in samples under both single drug aspirin and combinatorial DA therapy. Intracellular oxidative activity is a factor in metabolism and is a key regulating process for several core functions including cell proliferation and transcription.^65^ We further substantiated the drop in oxidative stress levels in cells under combination therapy using the peroxidase assay and MTT assay (Figure S16). Both MTT assay and peroxidase assay reveals a reduction in absorbance levels for cultures under combinatorial treatment even within 72 h of treatment, which may become more significant with prolonged treatment periods.

We further speculated the possibility of combinatorial DA therapy acting through the formation of a new compound formed in the process. Although high-performance liquid chromatography (HPLC) analysis of a DA mix shows a co-elution of compounds 2.1 and 2.2, further MS, nuclear magnetic resonance (NMR) and Carbon, hydrogen, nitrogen, sulphur (CHNS) elemental analysis show that both compounds were similar to doxorubicin (m/z spectrum (M + H) + of compounds 2.1 and 2.2: 544.17, 544.18) and were likely to be impurities. Hence the therapeutic effects appear to be the result of the combination of aspirin and doxorubicin, instead of formation of a new compound (Figure S17).

## Discussion

The benefits of anti-cancer combinatorial treatments have been proposed and observed for more than five decades. Yet, there is no absolute formula that can be fully effective against this disease. Such issues are largely hampered by the lack of well-defined procedures to analyse the heterogeneous responses of cancer cells to treatment and the mechanisms by which these single drugs work under combination regimens. Here, we demonstrated the use of a microfluidic assay for the analysis of combinatorial drug treatment efficacy on cell clusters cultured in 3D. This approach seeks to provide an assay for the potential screening of novel combinatorial drug regimens on cancer cell lines, and for clinical CTC clusters that can be formed with liquid biopsies from patients before or during treatment administration.

Current literature, mainly from observational studies, is not able to warrant a firm recommendation of the use of aspirin for reducing cancer risk, owing to the inconsistent results. These inconsistencies could be due to unreported behaviour factors or pre-existing conditions, statistical power, cohort selection or varying drug exposure conditions.^25^ It will be challenging to initiate widespread human clinical trials to iron out these issues, owing to the need for a large cohort size and procedures required to allow prolonged monitoring over time. A more viable alternative will be to put future efforts into generating in vitro models and animal models under controlled conditions to evaluate the potential toxicity and efficacy of aspirin and to determine if the use of aspirin can outweigh these risks in a patient-specific manner. Furthermore, different efficacy dosages of aspirin have been reported for various cell types, ranging from 29.3 µM for chondrocytes^66^ to 7.3 mM for B-chronic lymphocytic leukaemia cells.^67^ As a single drug supplement after anti-cancer treatment, aspirin exerts its benefits only after a latent period of 3 years (Table S2). Hence, patients are administered a high dosage of aspirin continuously, presenting a risk of drug toxicity, bleeding complications or mortality.^68^ Current studies suggested that the anti-cancer impact of aspirin can be highly varied with dosage and duration of intake. This contrasts with other applications of aspirin, for example, the addition of aspirin to clopidogrel beneficial for coronary syndromes is independent of aspirin dosage.^69^ There are also conflicting reports, which demonstrate that different aspirin dosages can reduce prostaglandin E levels to the same degree,^70^ hence conferring similar chemopreventive effects. It remains a challenge to validate the actual efficacy of aspirin with current literature, which fails to reflect underlying pre-existing conditions, statistical power, cohort selection or variations in drug exposure parameters.^25^ Some studies involving breast cancer even reported an inverse association of aspirin with cancer chemoprevention.^71^

Here, we demonstrate a heterogeneous response in terms of killing efficacy and CSC reduction, using clinical CTC cultures from liquid biopsies. CTC cultures were obtained efficiently by the cluster assay (60.9%), with cluster-forming potential varying with treatment time points to reflect patient prognosis.^72^ We confirmed the enhanced cancer-killing effect (66.7%) and reduction of CSCs (61.1%) under combinatorial treatment for clinical CTC cultures, albeit outcomes were heterogeneous. Among the pre-treatment samples processed (*n* = 6), better response under combinatorial DA treatment was observed in samples from patients with the ER + PR-HER− tumour subtypes. Heterogeneity in clinical outcomes was widely reported in other drug studies, even in recent PD-1 targeting immunotherapy trials.^73^ Nonetheless, these results present an intriguing correlation between heightened drug efficacy under combinatorial DA treatment for subpopulations of breast cancer, and a larger set of clinical samples is currently being collated to validate these findings.

The main mode of action of aspirin is mediated through the inhibition of COX enzymes and subsequently their key effectors, such as PGE2. The reduction of prostaglandins, which are active lipid compounds involved in proliferation, immunity and associated with cancer metastasis,^74^ has been demonstrated in colorectal cancer. The beneficial effects on patients with coronary syndromes appear to act through these mechanisms.^69^ Specifically, COX-1 is one of the two isoforms that is irreversibly inactivated by aspirin and is part of the core mechanism involved for anti-clotting effects of platelets^49^ even at low dosages (< 650 mg/day). However, its anti-cancer mechanisms remain under debate, in part due to tumour heterogeneity, along with the complexity and multiplicity of aspirin’s biochemical mechanisms. The inhibition of COX enzymes also affects several pathways, resulting in wide-ranging effects such as the inhibition of platelet aggregation and prevention of cardiovascular disease occurrence.^75^ Thus far, the anti-cancer effect of aspirin has always been explored as a single drug supplement and administered post-anti-cancer treatment. Furthermore, the intake of aspirin does not seem to confer consistent therapeutic benefits across different cancer types (Table S3).^19^ Several hypotheses have been suggested, such as the involvement of PIK3CA, which mediates PTGS2 expression, the gene that codes for COX-2.^76^ COX-2 inhibitors are also known to independently demonstrate potent anti-tumourigenic effects.^77,78^

We suggest that the anti-inflammatory effect of aspirin is exerted via inhibition of COX enzymes.^48^ DNA-damaging agents such as doxorubicin are known to induce expression of COX-2, in association with p53.^79^ The anti-cancer effects of our combinatorial treatment regime appear to be mediated by both COX-dependent and COX-independent pathways, as evident by reduction of COX-2 expression after combinatorial DA treatment. COX-independent pathways are also involved as the low doses of aspirin (< 500 mg/day) used here has little or no activity against COX-2.^80^ We propose that the reduced tumour-forming potential was also mediated by the suppression of inflammatory-associated signalling pathways involved in EMT, as evident by the concurrent reduction of mesenchymal marker expression.

We observed a reduction in COX-2 expression under combinatorial DA treatment, albeit more apparently at low aspirin dosages (Fig. 6d, e). We are motivated to work with lower doses of aspirin ( ≤ 500 mg/ml) under short-term treatment and controlled conditions, as patients under high dosage intakes often incur adverse reactions, such as bleeding.^68^ In anti-cancer studies, it was shown that low doses of aspirin ( < 500 mg/day) has little or no activity against COX-2,^80^ an observation that was also reflected in our studies (Fig. 6e). Previous studies also explored the use of COX-2 inhibitors on reversal EMT and tumour growth inhibition in bladder cancer, but only one of three drugs tested demonstrated the desired anti-cancer effects.^81^ Thus, low-dose aspirin in combination with doxorubicin may promote anti-cancer properties via multiple processes dependent and independent of the COX pathway.

EMT transition is regulated by a myriad of signalling pathways. One of these is known to be triggered during inflammation and cancer and mediated by IL-6 through Janus kinase–signal transducer and activator of transcription 3 (STAT3)-induced SNAIL1 expression.^55^ We also evaluated gene levels of STAT1 and STAT2 expression, known as key mediators of the classic host immune defense system (Figure S14) and noted the highest increase in STAT2 gene expression under combinatorial DA treatment as compared with other conditions. This suggests that the receptors of type 1 and type 3 IFNs may be involved, as STAT2 alone works via this pathway^82^ and has a unique role in cytokine signalling.^83^

Furthermore, it is important to study EMT phenotypes as specific subtypes such as the mesenchymal cells and CSCs are associated with key aspects of tumour progression.^39,84,85^ While high levels of IL-6 are commonly associated with the tumour microenvironment, IL-6 has also been reported to demonstrate anti-inflammatory properties and play important roles in metabolism.^58^ Disruption of IL-6 gene had been shown to result in increased systemic inflammation and reduced glucose tolerance.^61^ Recent findings have also suggested crucial roles of systemic immunity and cytokine-dependent homoeostatic programmes in the effectiveness of immune-based therapeutic methods^86^ and tumour progression.^87^

This maintenance of IL-6 expression levels with combinatorial DA treatment could generate less ‘stressful’ stimuli post treatment, which are known to have either a prodeath or prosurvival role and could drive cancer cells to become more metastatic and drug-resistant.^5^ Such observations suggest that the combinatorial DA therapy induces similar suppression of inflammatory signalling pathways while reducing therapeutic stress to prevent the onset of cellular adaptations, which will lead to more malignant subtypes via EMT.

Our current hypothesis argues that the effects of the combinatorial DA treatment are likely to involve both COX-dependent and COX-independent pathways, the former being evident by the reduction of COX-2 expression. Studies have demonstrated that COX-2/PGE2 pathways are potent inhibitors of EMT for epithelial cells,^31^ and the resultant COX-2-derived PGE2 and PGD2 are mediators of anti-EMT.^32^ Due to the varied outcomes under treatment with different aspirin concentrations, we propose that the combinatorial therapy with low-dose aspirin may also act via COX-independent pathways^88^ (Fig. 1c). This is within expectations as low-dose aspirin has been reported to have no effects on COX-2 expression,^80^ and various factors affecting COX expression are also reported to be involved in pathways that directly affect cancer cell behaviour (e.g., hypoxia-inducible factor-1α^89^). In fact, transforming growth factor-β1, a multifunctional cytokine involved in various pathophysiological processes, has been reported to induce downregulation of COX-2 while independently facilitating EMT in both normal and lung cancer cells.^90,91^ Here, we suggest that combinatorial DA treatment is also mediated by similar anti-inflammatory pathways, which lead to less ‘stressful’ stimuli post treatment. Such stimuli are known to have a prodeath or prosurvival role and drive cancer cells to respond or adapt to become more metastatic and drug-resistant.^5^ Our method of combinatorial DA treatment hence, in turn, could reduce the onset of metastatic phenotypes. Conferring anti-cancer benefits via COX-independent pathways is of certain benefits, as the excessive suppression of some anti-carcinogenic PGEs^92^ through the COX-dependent pathway may induce detrimental effects that are pro-tumourigenic instead, an effect likely to be dose-dependent as well.

Overall, our findings serve as a basis for optimism regarding the utilisation of low-dose aspirin in combination with anti-cancer drugs as an effective chemopreventive therapy against breast cancer and potentially other cancer types.

## Materials and methods

### Fabrication of gradient generator and liquid barrier layer

The device is the assembly of three functional layers, the bottom one containing the rounded ellipsoidal wells, the middle one with the open channels for liquid containment and the top one with the gradient generator microchannels. Each layer was produced separately in polydimethylsiloxane (PDMS), using a soft lithography approach. They were then aligned and permanently bonded via surface activation of the PDMS by O_2_ plasma.

The detailed description of the fabrication protocol was discussed elsewhere.^72^ In brief, the gradient generator and open channels layers require for standard photo-lithography (SU-8 negative tone photo-resist on a silicon wafer as substrate) for the former and metal micro-machining the latter to produce their respective primary molds. Two inlets allow the flow of a single drug or double drug combinations into the device (Fig. 1b), which will be mixed by the gradient generator component to achieve various drug combinations within the compartmentalised channels. Numbers of PDMS replicas could then be fabricated easily.

### Fabrication of 8-channel and 10-channel cancer cell cluster assays

The fabrication of the primary mold for the rounded wells was more complex. The size (250 µm major axis and 150 µm minor axis) and depth required (150 µm) does not allow for the use of a reflow strategy,^93^ due to lack of suitable photoresists that could be coated with the required thickness. Hence, we applied a diffuser back-side lithography procedure,^94^ which works using SU-8 negative tone photo-resist. SU-8 can be coated up to 1 mm thick and it results in more durable structures being negative tone.

Back-diffuser lithography works by exposing to UV-light a thick photo-resist coated on the surface of an optical mask from the back and through an optical diffuser, such as an opal diffuser plate. In this way, it is possible to define rounded volumes of exposed photo-resist. After post-baking and development, ellipsoidal pillars of hardened SU-8 remain on the mask plate and could be used as a primary mold for soft lithography. The number and distribution of these lenses were defined by the openings in the optical mask, therefore the choice of the number of channels and the density of wells for each channel can be freely defined.

Instead of using the optical plate directly as a primary mold, with the purpose of increasing its lifetime we choose to use a double-replica strategy. The first replica made in PDMS out of the optical plate was with ellipsoidal wells (same polarity as in the final device), but a second replica out of this PDMS mold was with ellipsoidal pillars, same as in the original optical plate. This last PDMS replica was finally used as a working mold for the fabrication of multiple layers with wells for the final device application.

### Cell line cultures

MDA-MB-231 (HTB-22TM, ATCC, USA), a human breast adenocarcinoma cell line, was used in the formation of cancer cell clusters. SW480 (ATCC CCL-227), a colorectal epithelial cell line was used to characterise cluster formation. Cell lines were cultured in high-glucose Dulbecco’s modified Eagle’s medium (DMEM) (Invitrogen, USA) supplemented with 10% fetal bovine serum (Invitrogen, USA) and 1% penicillin-streptomycin (Invitrogen, USA). Cultures were maintained under normoxia at 37 °C in a humidified atmosphere and 5% (v/v) CO_2_. Cells were cultured in sterile 25 cm^2^ flasks (BD Bioscience, USA) and sub-cultivated two times a week, with media refreshed every 48 h.

### Establishment and evaluation of CTC clusters from liquid biopsies

Sixty-nine blood samples collected from 45 patients with breast cancer (Table S3) using EDTA-coated vacutainer tubes (Becton-Dickinson). Blood cells and cell lines were controls for components of the clinical patient samples, mainly white blood cells and cancer cells respectively. Within 10 h, the blood samples were lysed with 2 × red blood cell lysis buffer (G-Biosciences, MO, USA, cat. no. 786–849). Intact non-RBCs were then removed by a centrifugation step at 1000 rcf for 5 mins. The resulting cell pellet was resuspended in 5 ml of 1 × phosphate-buffered saline (PBS) (Vivantis Inc., USA, cat. no. PB0344-1L) and centrifuged at 1200 rpm for 3 mins. The supernatant was discarded, and the cell pellet was resuspended in 1 ml DMEM (Thermofisher Scientific, cat. no. 11965118). Cells were quantified using an automated cell counter (Bio-Rad, CA, USA, cat. no. 786–849). A total of 2.0 × 10^7^ cells were seeded in each channel of the device to allow comparison of cluster-forming potential across samples. Samples were cultured for 14 days under a hypoxic condition at 37 °C, with 300 µl media change every 48–72 h. At the end of 14 days, phase-contrast imaging of samples at 100 × magnification was performed. Percentage of cluster formation was determined using Image J (Table 2). Samples with > 50% wells that contained clusters were considered positive. The remaining cultured channels were treated with a combination of 50% doxorubicin (Sigma-Aldrich, MO, USA, cat. no. D1515) and 50% aspirin (Sigma-Aldrich, MO, USA, cat. no. A5376) or 50% doxorubicin, respectively, for 72 h.

### Preparation for cell seeding

Cell monolayers were dissociated at 80% confluence using 0.01% trypsin and 5.3 mM EDTA solution (Lonza, Switzerland). Cells were seeded at a concentration of 250–500 cells per microwell for rapid establishment of cancer cell clusters.

### Maintenance of cultures on-chip

After cell seeding, the assay was placed in a humidified 150 mm dish and incubated under conditions stated above. Media was refreshed every 48 h or earlier depending on seeding concentration. The media could be replaced manually or with a syringe pump.^72^

### Quantifying the proportion of microwells with cluster formation

Cluster phenotype was detected with a macro ran on the ImageJ processing software (Figure S18). Microwells with denser cell packing reflect lower grey values, which will be detected as clusters. Grey values of two regions from each image were obtained: (1) Background (non-microwell) and (2) Cell region (within microwell), using an image processing software (ImageJ). In microwells with clusters, groups of grey scale values obtained within the cell region will be < 50% of the average grey scale value computed from the background region, demonstrating the presence of a denser region.

### Procedures for drug-screening

Doxorubicin (Sigma, cat. no. D1515) and Aspirin (Sigma, cat. no A5376) were diluted in 100% DMSO and stored as aliquots under − 20° C. Working concentrations of drugs were freshly prepared before use. Drugs could be introduced after overnight incubation or once clusters were established. For cultures beyond 72 h, drug solutions diluted in supplemented media were replaced every 72 h for up to 7 days of exposure.

For evaluating IC50 values of monolayer cultures, cells were seeded at 0.5 million cells per chamber of an eight-well plate (Ibidi GmbH, Germany) and exposed to a gradient of drugs added manually (Figure S1).

For screening of cancer cell clusters under various drug combinations, drugs could be introduced manually or via the gradient generator component of the assay. For a generation of drug concentrations via gradient generator, working concentrations of aspirin (500 mg/ml), doxorubicin (0.5 µM) and DA (0.5 µM doxorubicin + 500 mg/ml aspirin) were prepared, respectively, for syringe loading. Loaded syringes were connected to the assays via designated inlets. The media in the assay channels (250 µl for the 8-channel assay and 150 µl for the 10-channel assay) were gradually replaced with a continuous influx of fluid from the syringes pumped at 100 μl/min.

### Immunostaining of cancer cell clusters in situ

The viability of cells in each channel was determined with dyes that stain for live or dead cells, specifically Calcein AM (green, 2 µM; Life Technologies) and Sytox (far red, Thermofisher). Ethidium bromide was not used to avoid fluorescence emission overlap with doxorubicin. Clusters were incubated for 30 mins in situ under incubation. Alternatively, samples could be stained as single cells after gentle release from microwells by pipette resuspension. Imaging was carried out with an Olympus inverted confocal microscope (Emission filters ET460/50 m and ET535/50 m; Olympus, Tokyo, Japan). Images should be obtained with the appropriate filters to reduce background signals from doxorubicin. Cells counts were obtained using ImageJ via thresholding (NIH, Bethesda, MD).

For immunostaining with other antibodies, clusters were released from the microwells and resuspended as single cells for analysis. For membrane proteins, samples were stained directly with fluorochrome-conjugated antibodies (CD44-fluorescein isothiocyanate (green; FITC) and CD24-Allophycocyanin (red; *APC*) (1:100, Miltenyi Biotec Asia Pacific, Singapore). For cytoplasmic proteins (Caspase-3; 1:250, cat. No. ab13847, Abcam) (pan-cytokeratin-FITC; 1:100, Miltenyi Biotec Asia Pacific, Singapore and (Vimentin; 1:250, DAKO), samples were fixed with 70% ethanol for 10 min and stained with their respective primary antibodies, washed and stained with the appropriate secondary antibodies (anti-mouse or anti-rabbit Alexa Fluor® 488, Abcam and Alexa Fluor® 633, Abcam). Samples were incubated in the antibody cocktail for an hour, washed with PBS and imaged with confocal microscopy.

### Profiling viability proportions

To establish the IC_50_ values, z-stacks images of 25 microwells from each channel were obtained. Images from each stack were merged based on maximum intensity. Merged images were processed to identify maxima signals corresponding to each cell. For consistency, the microwells considered for evaluation were obtained at the same distance from the assay inlets.

### Profiling CSC-like proportions

Images were obtained with confocal microscopy and processed with the ImageJ processing software. Specifically, locations of CD44^+^ and CD24^+^ cells were identified by setting a 'threshold' using ImageJ’s thresholding tool. Twenty-five fields were evaluated per set (~ > 15,000 cells). Processed images were merged to locate regions with overlapping CD44 and CD24 signals (i.e., CD44^+^/CD24^−^ cells). This proportion was then subtracted from the overall counts of CD44^+^ single positive cell populations to obtain CD44^+^/CD24^−^ cell populations corresponding to the CSC-like fractions. For clinical samples, CD45^−^/CD44^+^/CD24^+^ populations were identified as the CSC cohorts.

### Cluster and spheroid colony assays

Treated cultures were transferred as a single-cell suspension to a new microwell-based cluster assay to determine cluster-forming potential.^34^ Proportions of resultant clusters were evaluated as previously described. Similarly, for spheroid assays, single cells were mixed to a final concentration of 0.35% top agarose and added to a base layer of 0.5% solidified agarose. Spheroids were stained with Hoechst and imaged with an Olympus inverted confocal microscope (Olympus, Tokyo, Japan). All assays were maintained for 2 weeks prior to analysis.

### Evaluation of COX-1 activity levels

Cultures were lysed and homogenised by freeze-thaw with dry ice and sonication in cold buffer. COX activity was evaluated using a COX Activity Assay Kit (760151, Cayman Chemical). Samples were prepared as indicated by manufacturer’s instructions and readings were obtained with the Tecan i-control, software 1.10.4.0 (Tecan, Crailsheim, Germany).

### IL-6 ELISA procedure

Supernatants from untreated and treated cell culture were assessed for IL-6 secretion by ELISA assay (DY206; R and D systems) according to the manufacturer’s instruction. Basically, wells of 96-well microtitre plate were coated (overnight, room temperature) with 2 μg/ml of mouse anti-human IL-6 capture antibody in 50 μl of PBS. The plate was then blocked with 1% w/v bovine serum albumin in PBS for 2 h at room temperature. Standards and samples were then loaded in duplicates and incubated for 2 h at room temperature, followed by detection antibody (biotinylated goat anti-human IL-6) for another 2 h at room temperature. Streptavidin HRP was added for 20 min at room temperature and the reaction was visualised by the addition of 50 μl of TMB substrate for 10–20 min. The reaction was stopped with 25 μl sulphuric acid (H_2_SO_4_, 2n) and absorbance was measured at 450 nm with a reduction in 570 nm using Tecan Infinite M200 plate reader. The plate was washed three times with washing buffer (PBS with 0.05% (v/v) Tween 20) after each step. A standard curve was established using the absorbance gotten from a serial dilution of recombinant IL-6 (18.8 pg/ml – 2000 pg/ml). Absolute concentrations of the samples were calculated using Prism 7 (Graphpad Software Inc).

### Oxidative activity assays

The relative oxidative activity of cultures was measured with the MTT assay (Roche 11465007001) and peroxidase assay. Cultures were maintained and treated for 72 as previously described, before being harvested by gentle agitation. For the MTT assay, cells were reseeded into a well of a 96-well plate and incubated with the reagents provided as indicated by manufacturer’s instructions. In brief, measurements were obtained at 550 nm and 690 nm with an automatic microplate reader (Tecan Infinite M200), followed by analysis of the difference in absorbance levels with the Tecan i-control, software 1.10.4.0 (Tecan, Crailsheim, Germany). For the peroxidase assay, reactions were prepared as indicated by manufacturer’s instructions (cat no. MAK092, Sigma-Aldrich Ltd., USA) and analysed at 570 nm with the microplate reader and software. Each sample was tested in duplicate with the average of eight readings per well recorded.

### HPLC analysis and purification

The Atlantis Prep C18 reverse-phase column (Waters) was first equilibrated with 10% acetonitrile, 0.1% trifluoroacetic acid (TFA) in water at a flow rate of 5 ml/min for 15 min. 100 µl of sample was then loaded onto the column and the mobile phase was changed to 50% acetonitrile, 0.1% TFA in water under a linear gradient over a period of 55 min at a flow rate of 5 ml/min. For the next 5 min, a linear gradient to 100% acetonitrile containing 0.1% TFA was conducted. The eluted compounds were detected by measuring the absorbance at 270 nm and 475 nm. Desired fractions were collected, frozen in liquid nitrogen and lyophilised using a VirTis benchtop freeze dryer.

### Mass spectrometry

HPLC-MS analyses of the compounds were carried out on a 6550 iFunnel QTOF, connected with a 1290 UHPLC system (AGILENT, Singapore). The Zorbax Eclipse Plus C18, 2.1 mm × 100 mm column, with 1.8 µm particle size (AGILENT) was first equilibrated with 1% acetonitrile, 0.1% formic acid in water for 0.6 min at a flow rate of 500 µl/min. The samples were dissolved in 100 µl DMSO, and 5 µl was loaded for the analysis. Separation of the compounds was performed with a gradient of 1% acetonitrile, 0.1% formic acid in water to 99% acetonitrile, 0.1% formic acid in water for over 5 min. In total, 99% acetonitrile, 0.1% formic acid in water was then maintained for another 1 min, with the column temperature set at 40˚C throughout the run. The mass spectrometer was set to an MS scan range from 50 to 1000 m/z at 3 scans/sec. Data were recorded with Masshunter Acquisition B6.0 (AGILENT) and analysed with Masshunter Qualitative Analysis software version 6 (AGILENT).

After MS detection, CHNS elemental analyses were conducted by the Elemental & Thermal Laboratory in the Department of Chemistry, National University of Singapore. Elemental analysis was done using Vario MICRO cube elemental analyser, Elementar Analysensysteme GmbH, Hanau, Germany. NMR analyses were subsequently carried out by the Nuclear Magnetic Resonance Laboratory (School of Physical and Mathematical Sciences, Chemistry and Biological Chemistry department, Nanyang Technological University). ^1^H NMR spectra were performed using Bruker Avance III 400 (400 MHz). Chemical shifts (ppm) were recorded with a residual solvent peak as an internal reference.

### RNA extraction

RNA was extracted from cultures using the RNeasy Mini Kit (Qiagen, Germany). Cultures were lysed and homogenised with freeze-thaw and shear force cell disruption in buffer RLT as provided. Total RNA was eluted from the RNeasy Mini columns with 5 μl of RNase-free water. The rest of the procedure was performed according to the manufacturer’s protocol.

### Reverse transcription

In total, 1 μl of 50 μM oligo (dT) _20_ primers (Life Technologies) and 1 μl of 10 mM dNTP Mix (Life Technologies) were added to 5 μl of each lysed RNA sample. The sample was incubated at 65 °C for 5 min and subsequently cooled on ice for at least 1 min. In all, 1 × first-strand buffer, 5 mM DTT, 40 U RNaseOUT Recombinant RNase Inhibitor, 200 U SuperScript III RT (all Life Technologies) were added to a final volume of 20 μl. The following thermal setting was applied on a Verity 96-well Thermal Cycler (Applied Biosystems): 25 °C for 5 min, 55 °C for 60 min and 85 °C for 5 min. Each RT product was diluted to a final volume of 40 μl to avoid qPCR inhibition.

### Real-time quantitative PCR (RT-qPCR)

RT-qPCR was carried out in real-time using SYBR Green I detection chemistry on a Bio-Rad CFX96 Real-Time PCR Detection System (Bio-Rad Laboratories). Single-plex PCR was performed in a final volume of 10 μl, containing 300 nM of each primer (Integrated DNA Technologies), 1 × FastStart SYBR Green Master mix (Roche) and 1 μl of diluted RT product. Gene primer pairs used in this study were selected from PrimerBank (https://pga.mgh.harvard.edu/primerbank/) and are listed in Table S4. In silico PCR was confirmed with UCSC genome browser (https://genome.ucsc.edu/) for each primer set validation. All primer specificities were confirmed with a single peak during melting curve analyses. The following thermal setting was applied on the RT-qPCR Cycler: 95 °C for 10 min, followed by 40 cycles of amplification (95 °C for 20 sec, 60 °C for 30 sec and 72 °C for 20 sec) and a final additional incubation at 72 °C for 7 min. Gene expression data were normalised to two housekeeping genes (GADPH and UBB) with the following equation: relative expression = 2^−(*Cq*(*gene of interest*)−mean *Cq*(*housekeeping genes*))^. All measurements were performed in duplicate and mean values of relative expression are shown.

### Statistical analysis

All error bars represented standard deviation of triplicate cultures from different samples. Groups were compared using the Student’s *t* test to evaluate associations between independent variables and the *p* values were obtained using 0.01 or 0.05 significance levels, two-tailed. Adjusted multivariate analyses for continuous independent variables (to other variables) required larger sample sizes and were not utilised in this study. Resultant viability percentages were normalised to that obtained from samples in the last channel (lowest drug concentration). A four-parameter logistic equation was employed using Microsoft Excel® (Redmond, WA) for curve fitting analysis to determine IC_50_ values. The IC_50_ value was obtained as the concentration value at which the curve passed through the 50% normalised response value corresponding to the percentage of cell death (*y* axis).

### Availability of data and materials

The data sets supporting the conclusions of this article are stored in a secured research database and may be available upon presentation of formal approval.

## Electronic supplementary material

Supplementary File
