# Supplementary material for: Low-dose anti-inflammatory combinatorial therapy reduced cancer stem cell formation in patient-derived preclinical models for tumour relapse prevention
Source: Br J Cancer. 2019 Feb 4;120(4):407–23. doi: 10.1038/s41416-018-0301-9 (PMC6461953; doi:10.1038/s41416-018-0301-9)
Supplement: Supplementary file 1 — Supplementary File [file 41416_2018_301_MOESM1_ESM.docx]

**Supplementary Material**

**Supplementary Figures**

**
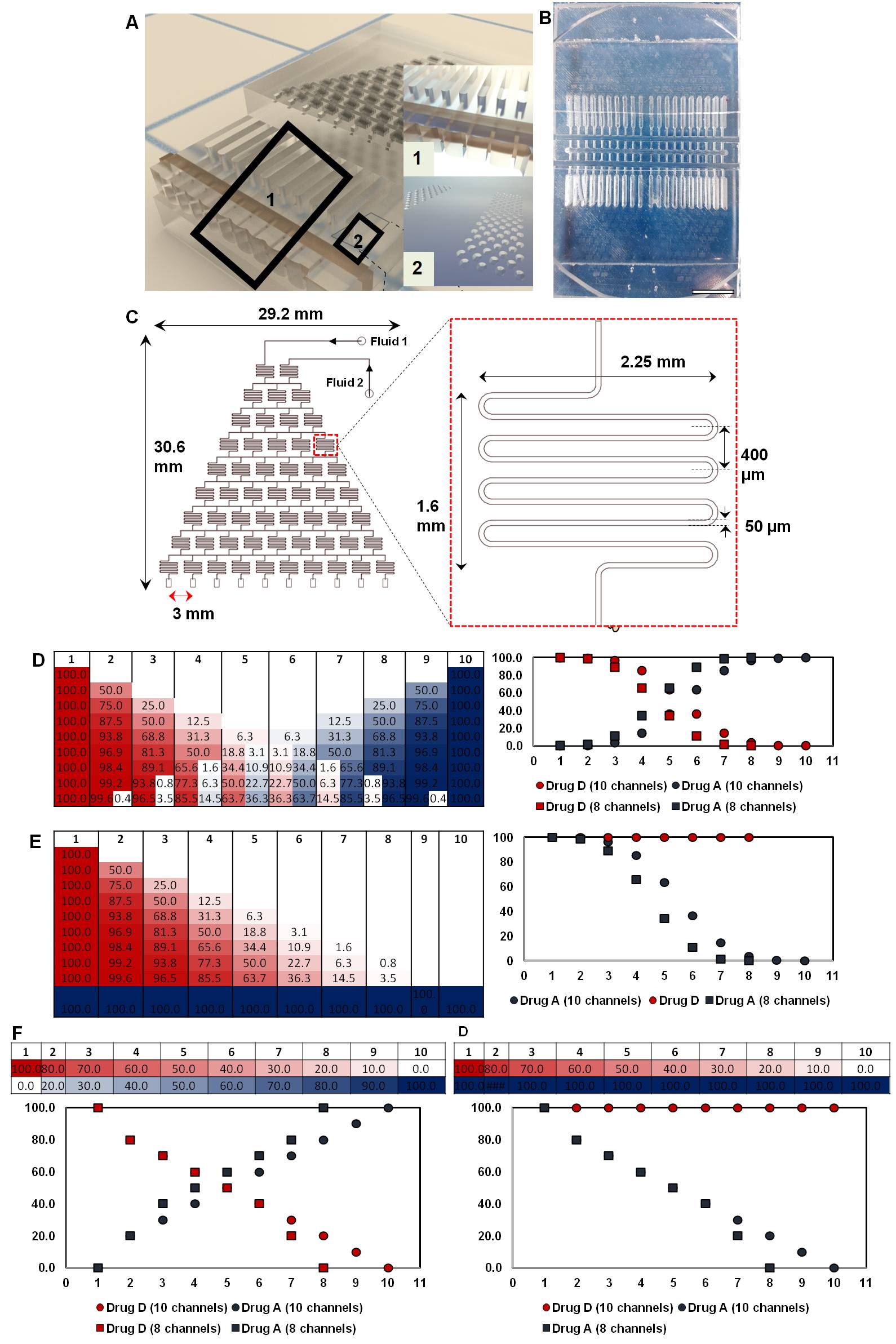
**

**Fig. S1 Device set-up with gradient generator.** (A) Details of the three functional layers. (Left). Schematics of valves for waste disposal (Inset 1). The bottom layer of the device contains the ellipsoidal wells (Inset 2), arranged in correspondence with the position of the open channels of the middle layer. (B) An actual microfabricated device. Scale bar is 5 cm. (C) Schematics of the gradient generator composed of two inlets each leading to “serpentine-like” micro-channels. The gradient of concentrations was generated by subsequently splitting the liquid exiting from one of such elements in two following elements of the same geometry. In the final step, the liquid collected at the output of the last portion of “serpentine-like” micro-channels were directed to the open channels in the middle layer of the device. The gradient of concentration entering the open channels was generated within the topmost layer of the device by splitting and subsequently mixing two different fluids fed into the two inlet ports. The “serpentine’-like” base elements of the gradient generator allows for the diffusion-driven mixing of two fluids introduced from the two inlet branches. In the downstream portion of the gradient generator, the exit from each element was finally released in the open channels. (D) Concentration gradients (n = 8 or n = 10) generated with different modes of drug exposure. Distribution of two drugs in combination with the effect of gradient generator. (E) Distribution of one drug in combination with another drug under fixed concentration. (F) Distribution of two drugs in combination with the manual addition of compounds for 2D cell cultures. (G) Distribution of one drug in combination by manual addition with another drug under fixed concentration.

**
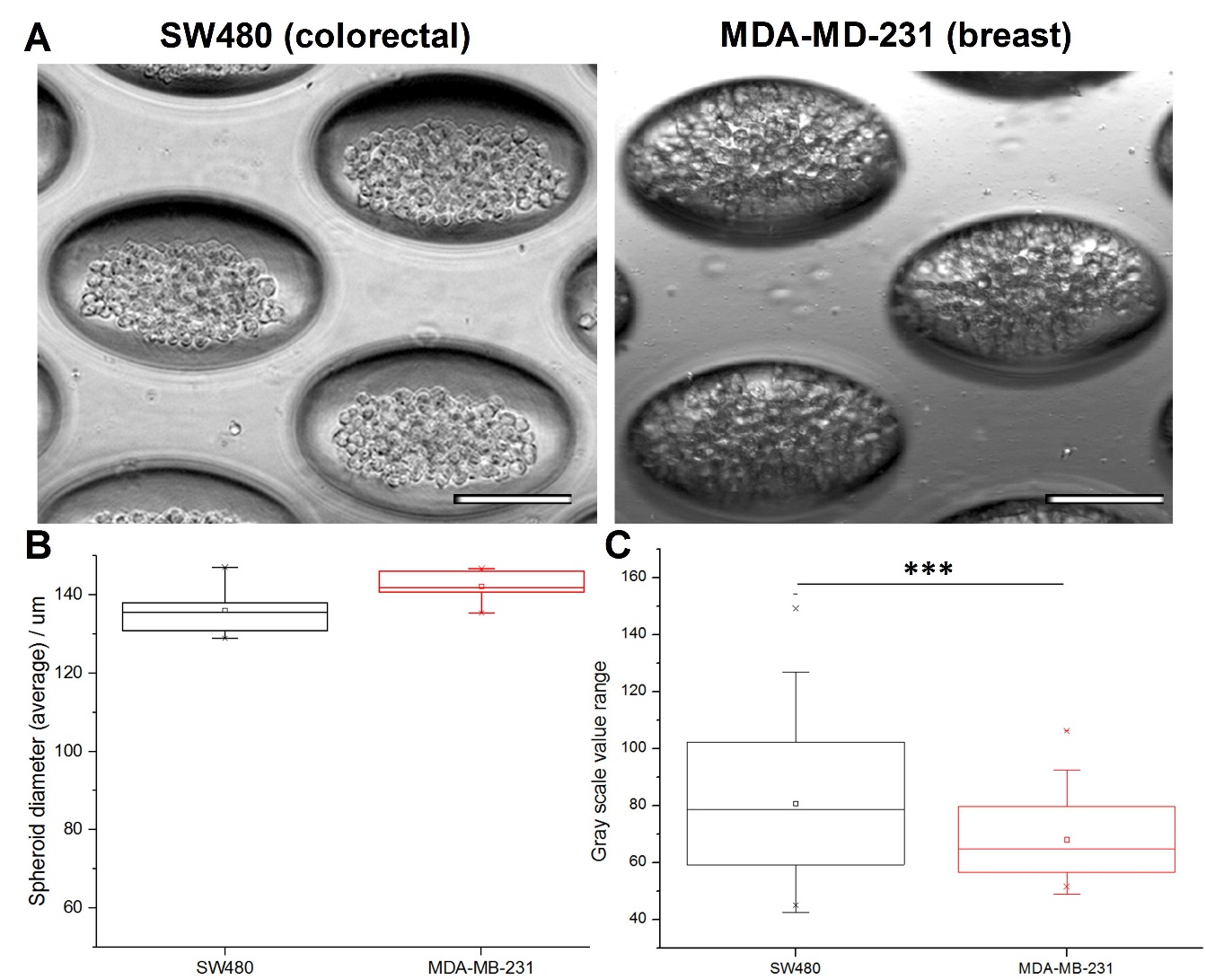
**

**Fig. S2.** Characterization of cluster formation. (A) Cluster formation with cell lines of different epithelial-mesenchymal transition (EMT) phenotype (SW480 and MDA-MB-231). Scale bar is 100 µm. (B) Box plot demonstrating consistent average diameter of clusters. (C) Box plot demonstrating cluster packing density in cell lines of different EMT phenotypes, i.e. cell types which demonstrated higher and consistent grey value ranges reflected closer cell-cell packing. *** *p* < 0.00001.


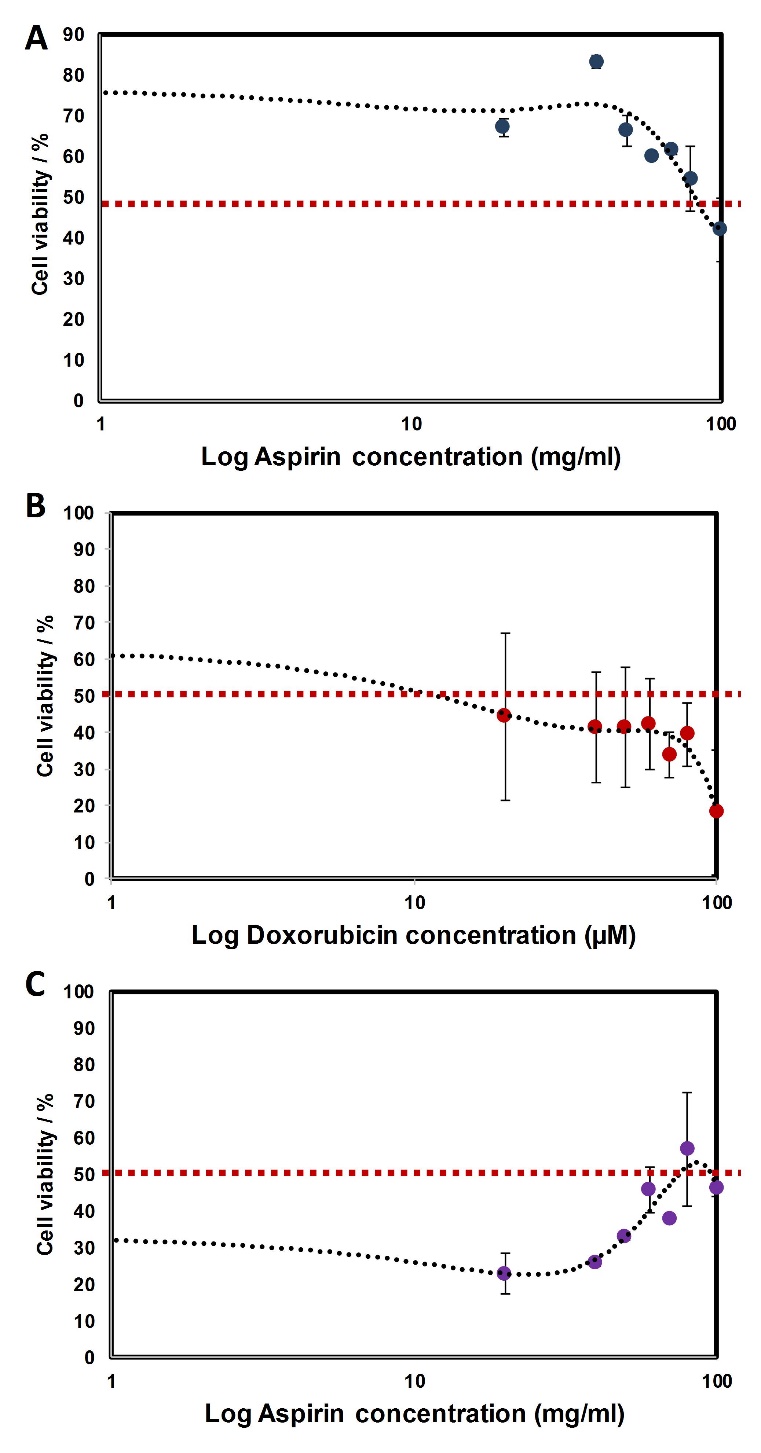


**Fig. S3 IC50 curves of 2D monolayer cultures of MDA-MB-231.** (A) Viability plot for cultures treated with aspirin only. (B) Viability plot for cultures treated with doxorubicin only. (C) Viability plot for combinatorial DA treated cultures, with varying concentrations of aspirin and fixed 0.5D. Values corresponding to 50% cell viability are marked with red dotted lines in their respective charts.


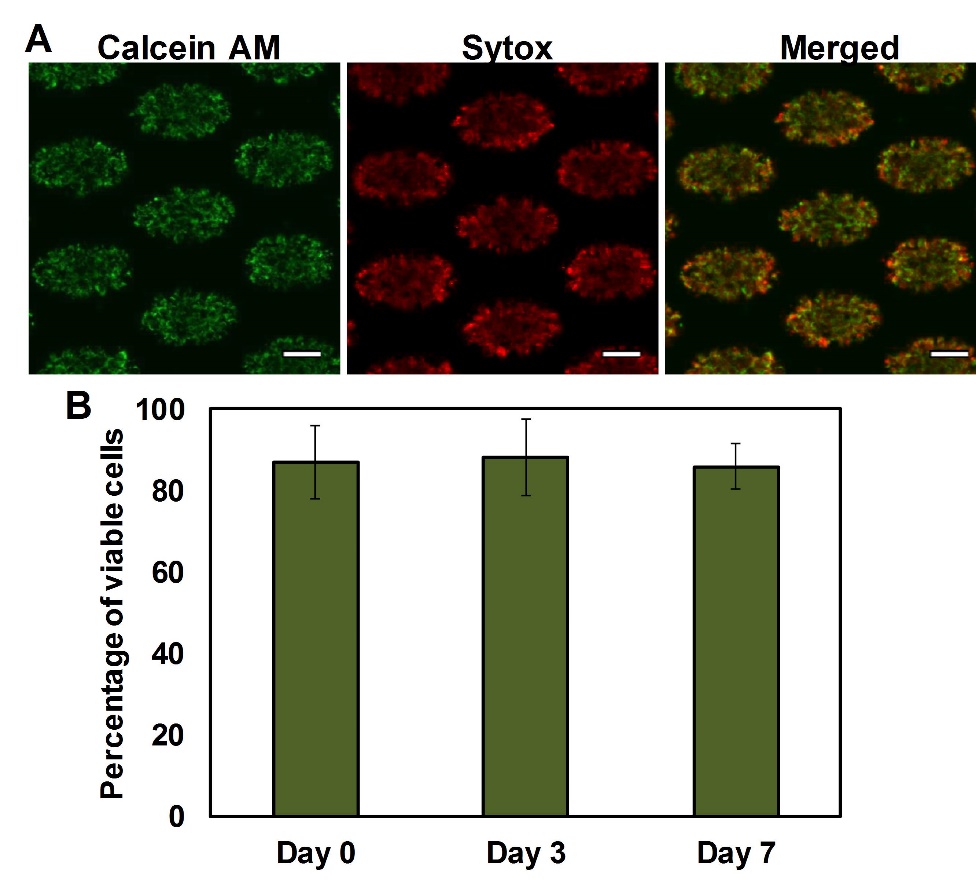


**Fig. S4**. **Assessment of cell viability and apoptosis**. (A) Representative images of breast cancer cell clusters under live/dead staining *in situ* using Calcein AM and SYTOX. Scale bar is 100 µm. (B) Percentage of viable cells in untreated cultures over time. No significant changes in percentages of viable cell populations are observed (*p* < 0.01).


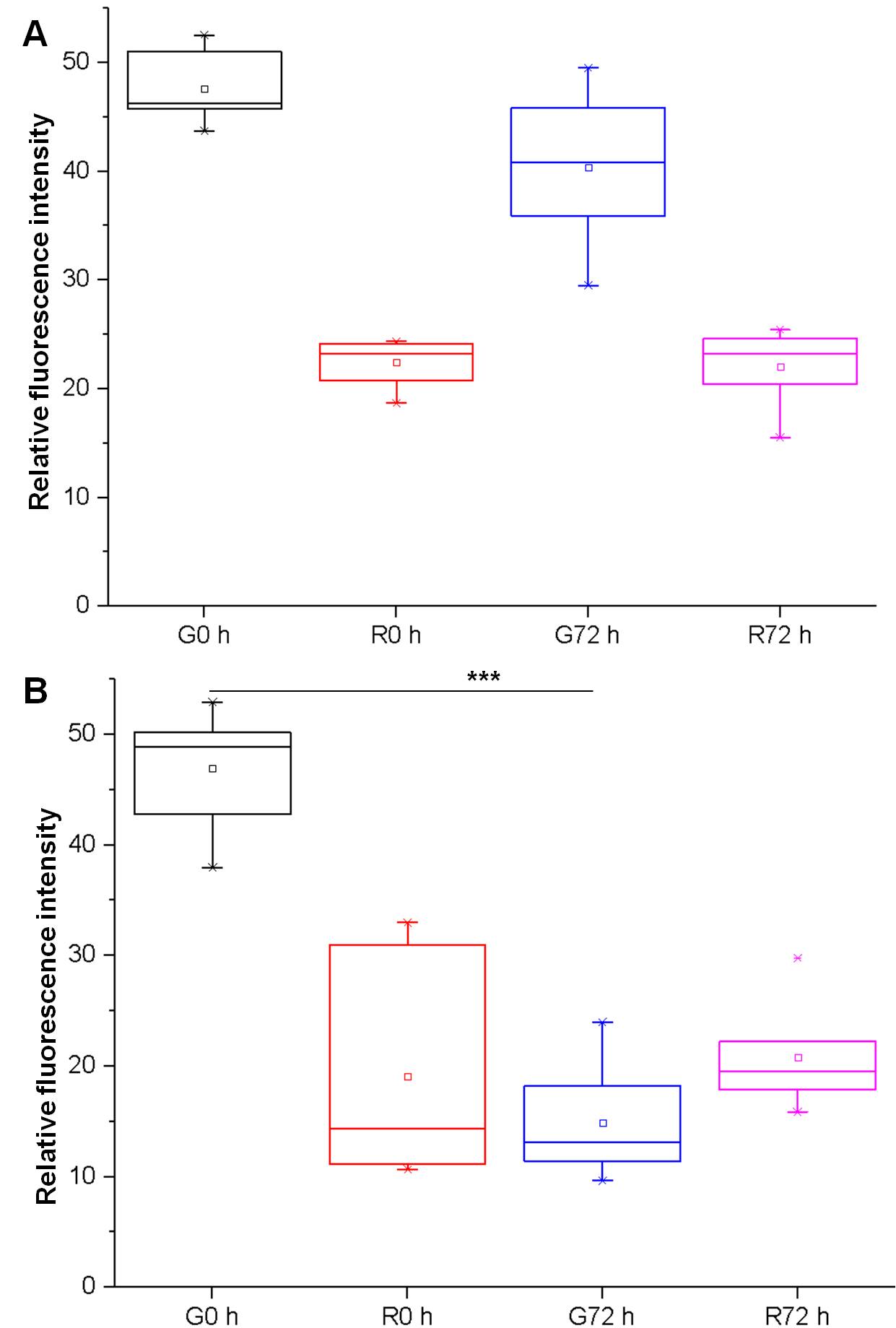


**Fig. S5 Quantification of relative fluorescence intensity for calcein AM and SYTOX.** (A) Fluorescence intensity levels for aspirin-treated cells at 0 or 72 h time points. G = Calcein AM. R = SYTOX. (B) Fluorescence levels for doxorubicin-treated cells at 0 or 72 h time points. G = Calcein AM. R = SYTOX.


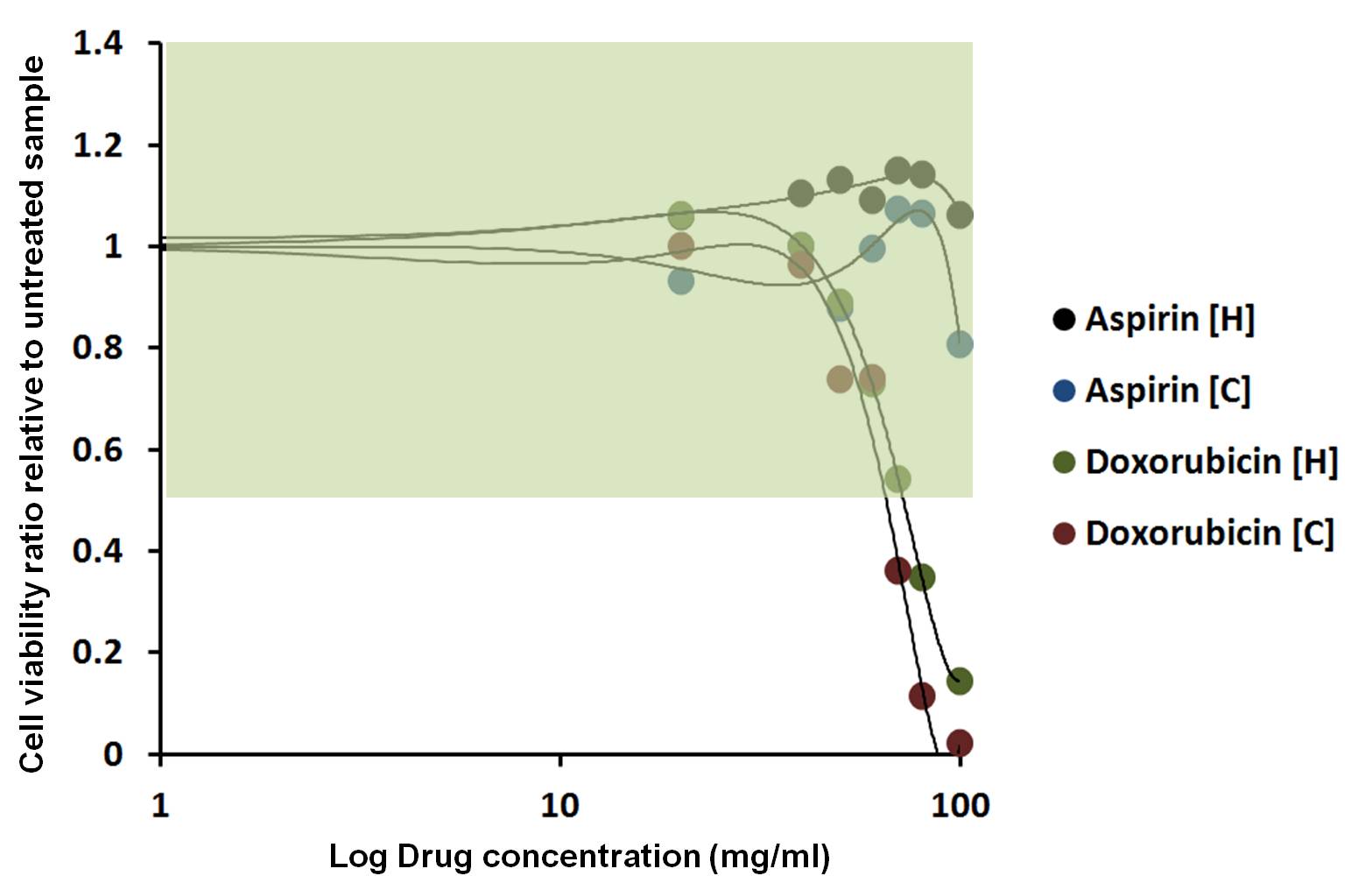


**Fig. S6 Merged dose-response curves of healthy and cancer samples under single treatment of aspirin or doxorubicin (n=3).** Marked regions show the concentrations omitted during evaluation of long-term cultures for subsequent drug experiments.

**
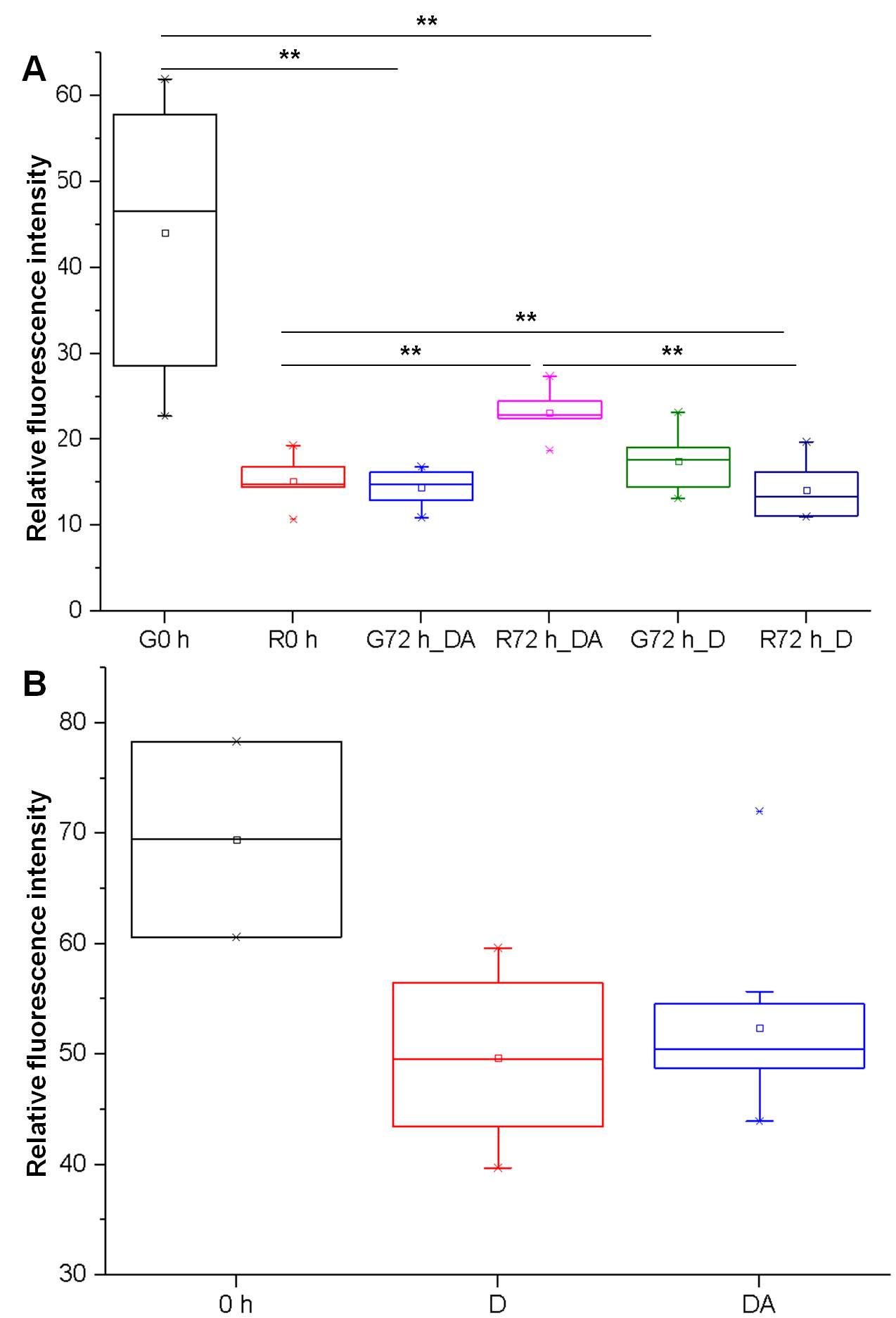
**

**Fig. S7. Quantification of relative fluorescence intensity.** Fluorescence intensity levels of calcein AM and SYTOX for cells before treatment (0 h) and doxorubicin-treated (D) or combinatorial treated (DA) cells at Day 7. G = Calcein AM. R = SYTOX. (B) Fluorescence intensity levels of caspase-3 in cells before (0 h) and after 7 days of treatment.


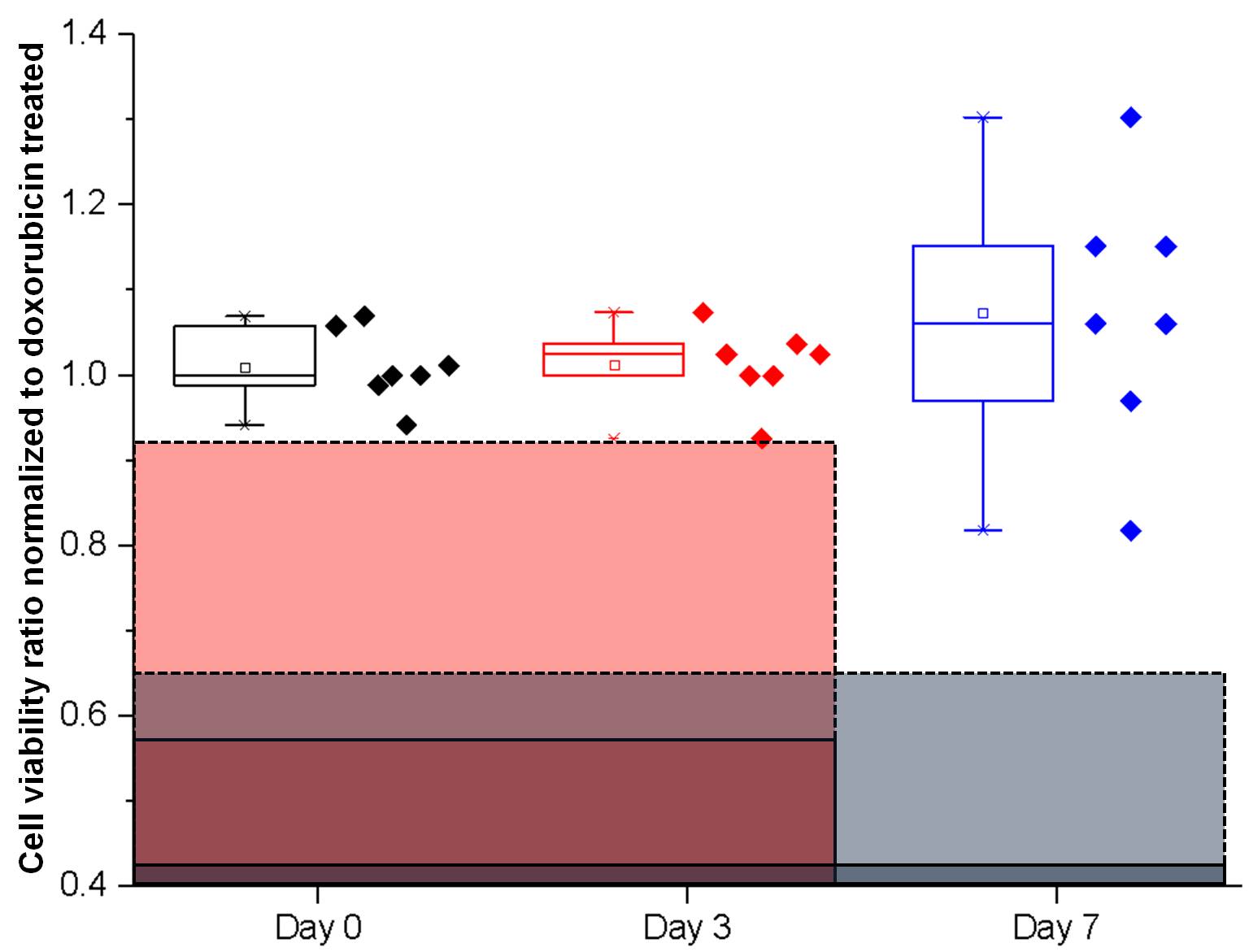


**Fig. S8. Comparison of reduction in viability ratio and CSCs over various treatment periods.** Y-axis denotes the viability ratio varied with aspirin concentration under combinatorial DA therapy, relative to that of cultures under doxorubicin treatment only. Boxed regions indicate the proportion of CSCs after 72 h treatment (red) or after 7 days (blue) treatment with combinatorial DA treatment respectively. Combinatorial DA treatment with low-dose (dashed, black) or high-dose (full, black) aspirin concentrations were screened accordingly.


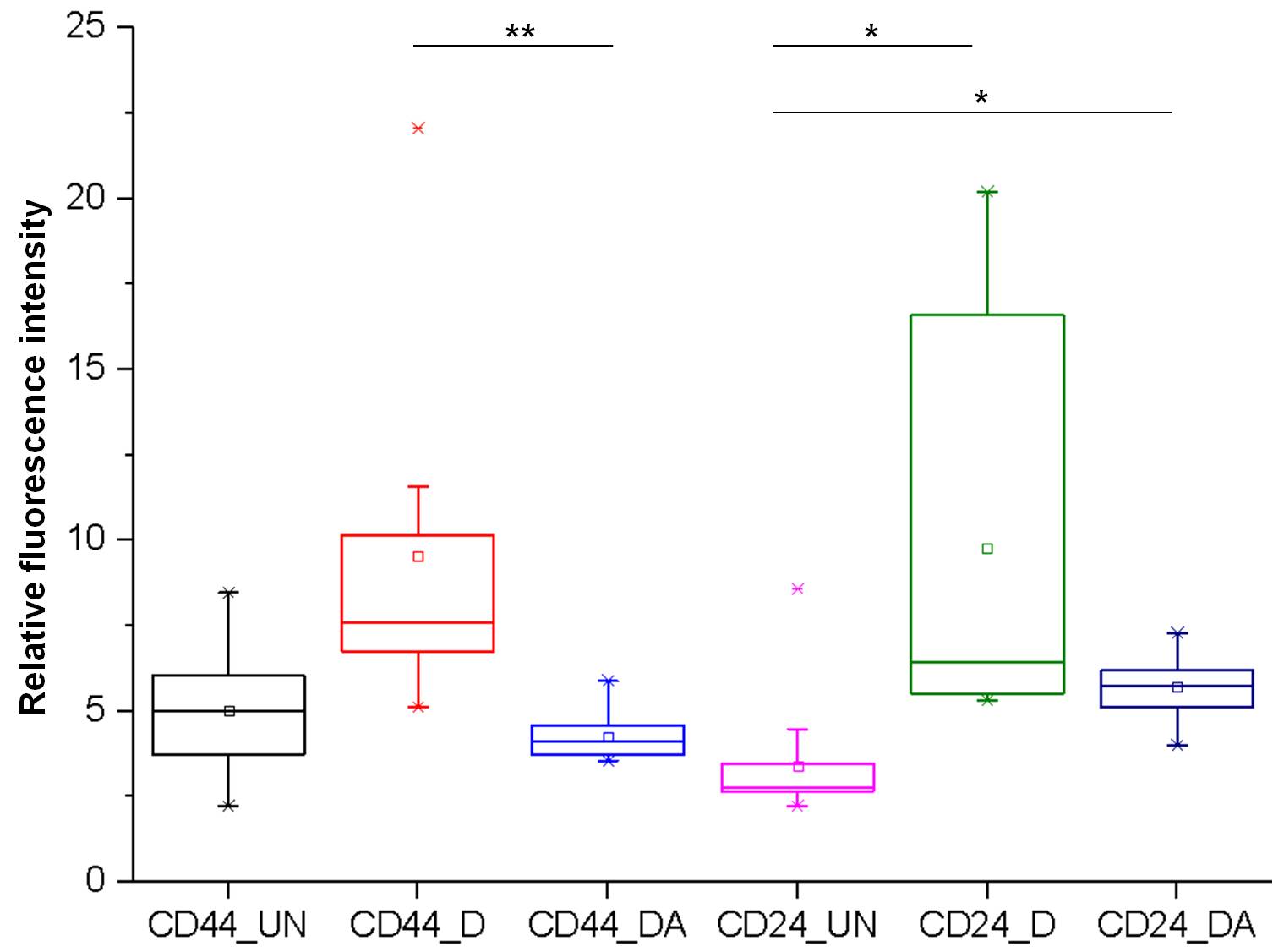


**Fig. S9.** **Quantification of relative fluorescence intensity.** Fluorescence intensity levels of CD44 and CD24 for cells untreated (UN) or after treatment with doxorubicin (D) or combinatorial treatment (DA) cells at Day 7.


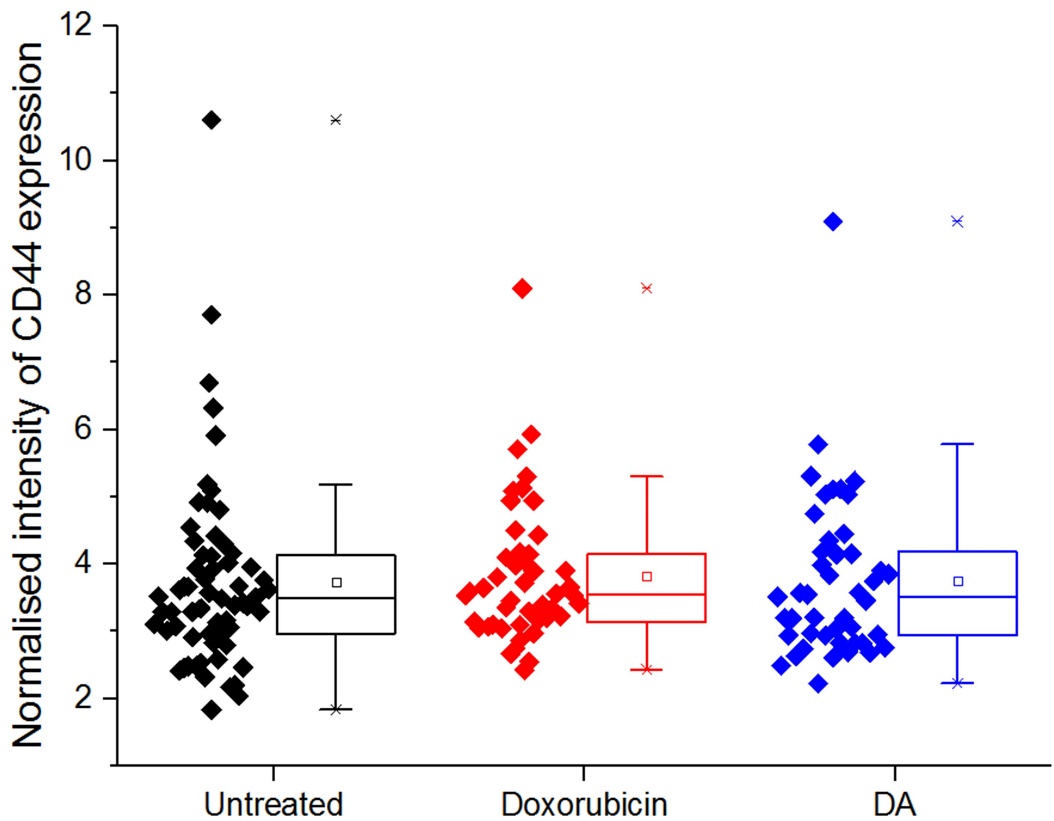


**Fig. S10 CD44 expression levels determined by fluorescence intensity, normalized to background values.** Doxorubicin concentration was 0.5 µM and combinatorial DA treatment was 500 mg/ml aspirin with 0.5 µM.

**
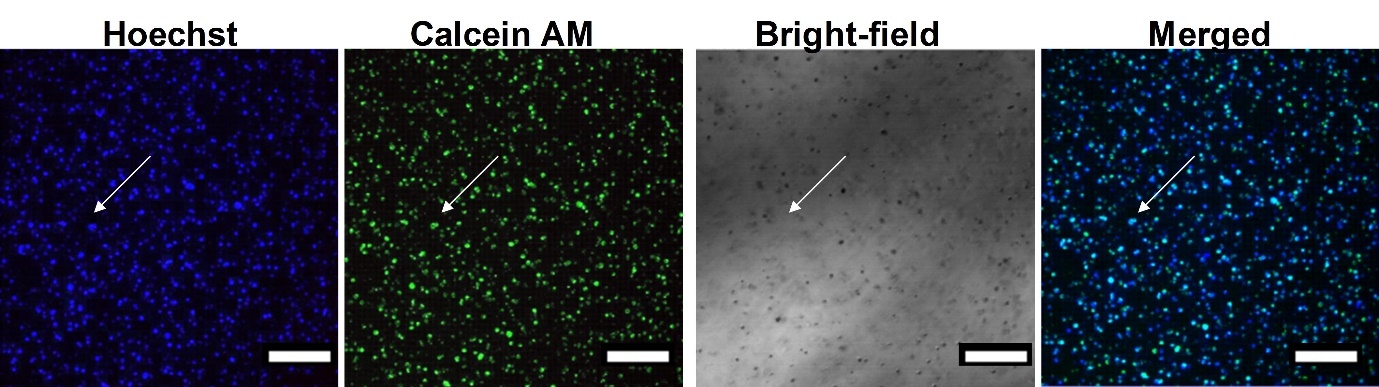
**

**Fig. S11 Representative images of a stained cancer cell cluster (Calcein AM/Hochest) in the gel.** Scale bar is 100 µm.

**
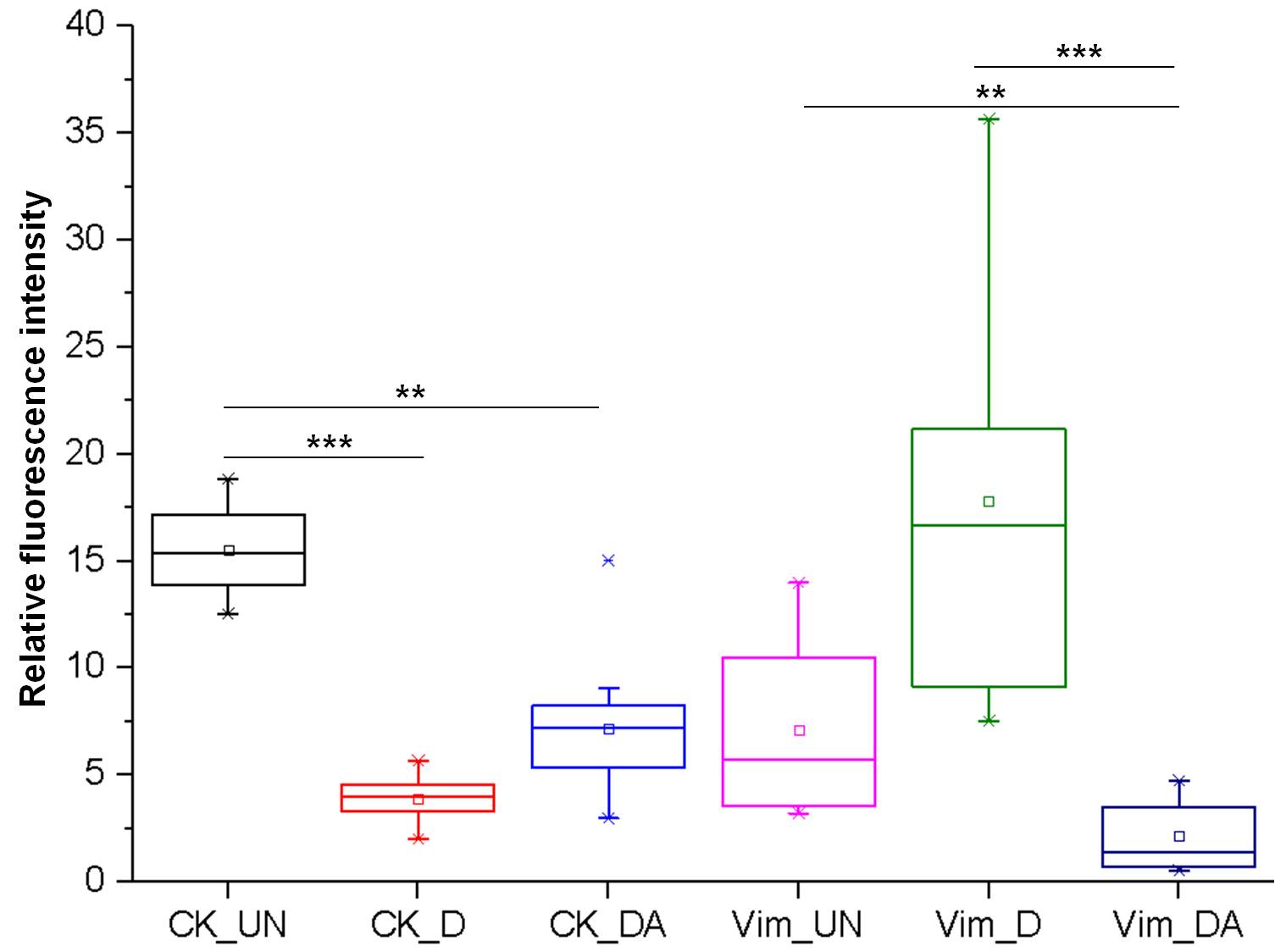
**

**Fig. S12 Quantification of relative fluorescence intensity.** Fluorescence intensity levels of cytokeratin (CK) and vimentin (Vim) for cells untreated (UN) or after treatment with doxorubicin (D) or combinatorial treatment (DA) cells at Day 7.

**
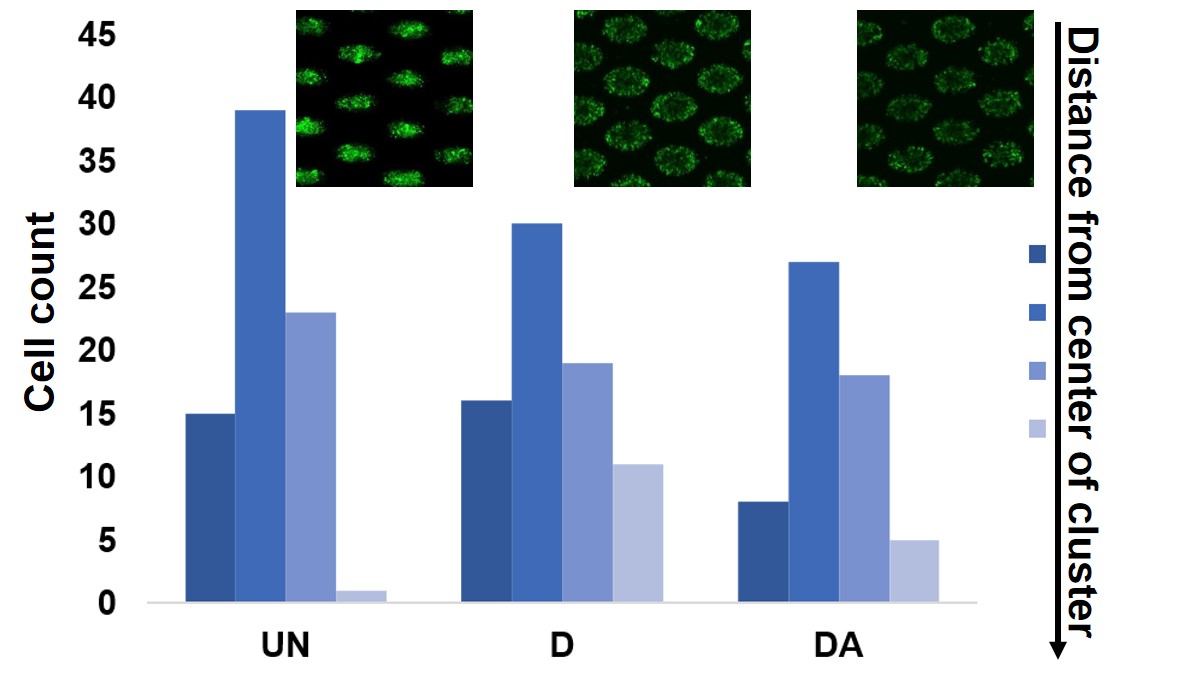
**

**Fig. S13 Relative distribution of cells within the cluster.** Each bar depicts cell count at differing distance (< 30 µm, < 60 µm, < 90 µm, > 120 µm) from the center of cluster.


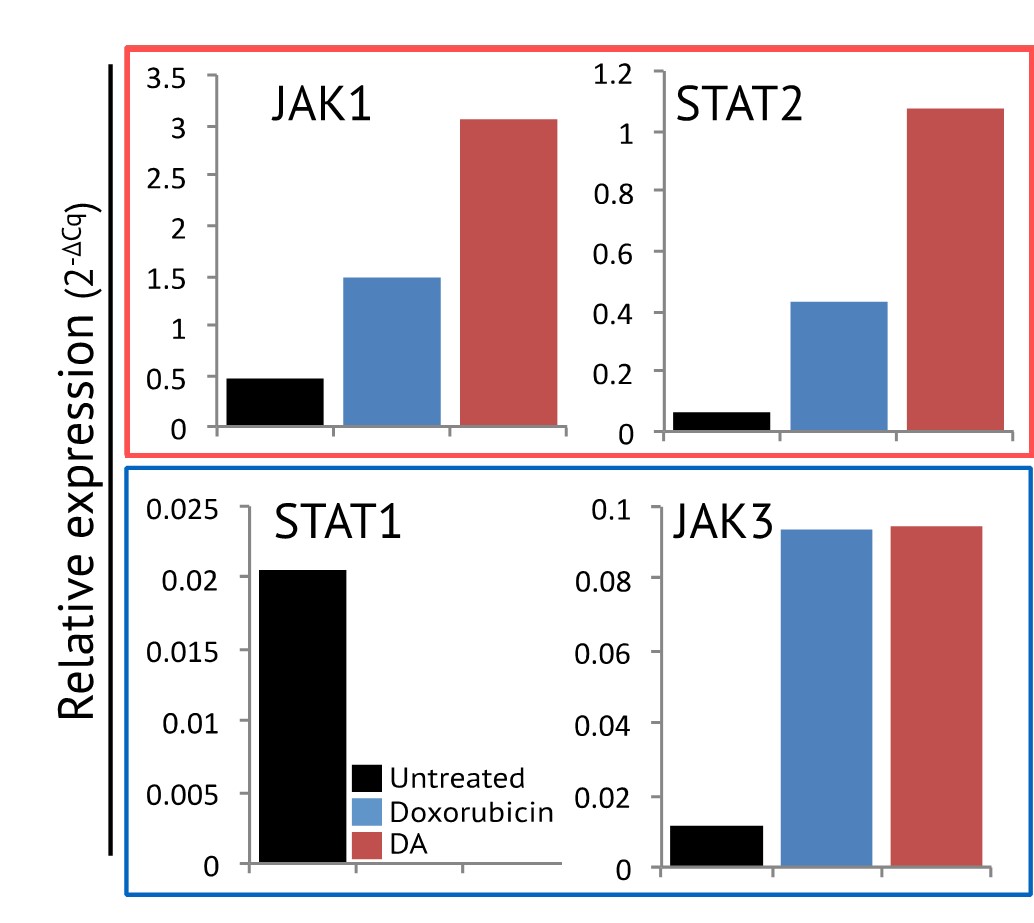


**Fig. S14. Pathway activated under combinatorial DA administration.** Expression of six genes (STAT1, STAT2, STAT3, JAK1, JAK2 and JAK3) were measured from two independent batches under three different conditions (untreated, doxorubicin-treated, and DA-treated samples). All values were normalized to two housekeeping genes (GAPDH and UBB) and were averaged from duplicates for each relative gene expression. The red borderline box highlights the genes (JAK1 and STAT2) with a progressive increase in expression under drug treatment. STAT1 was not expressed in drug-treated samples, whereas JAK3 expression was increased in samples treated with doxorubicin alone and combinatorial DA (blue borderline box). JAK2 and STAT3 were not expressed in any of the conditions.


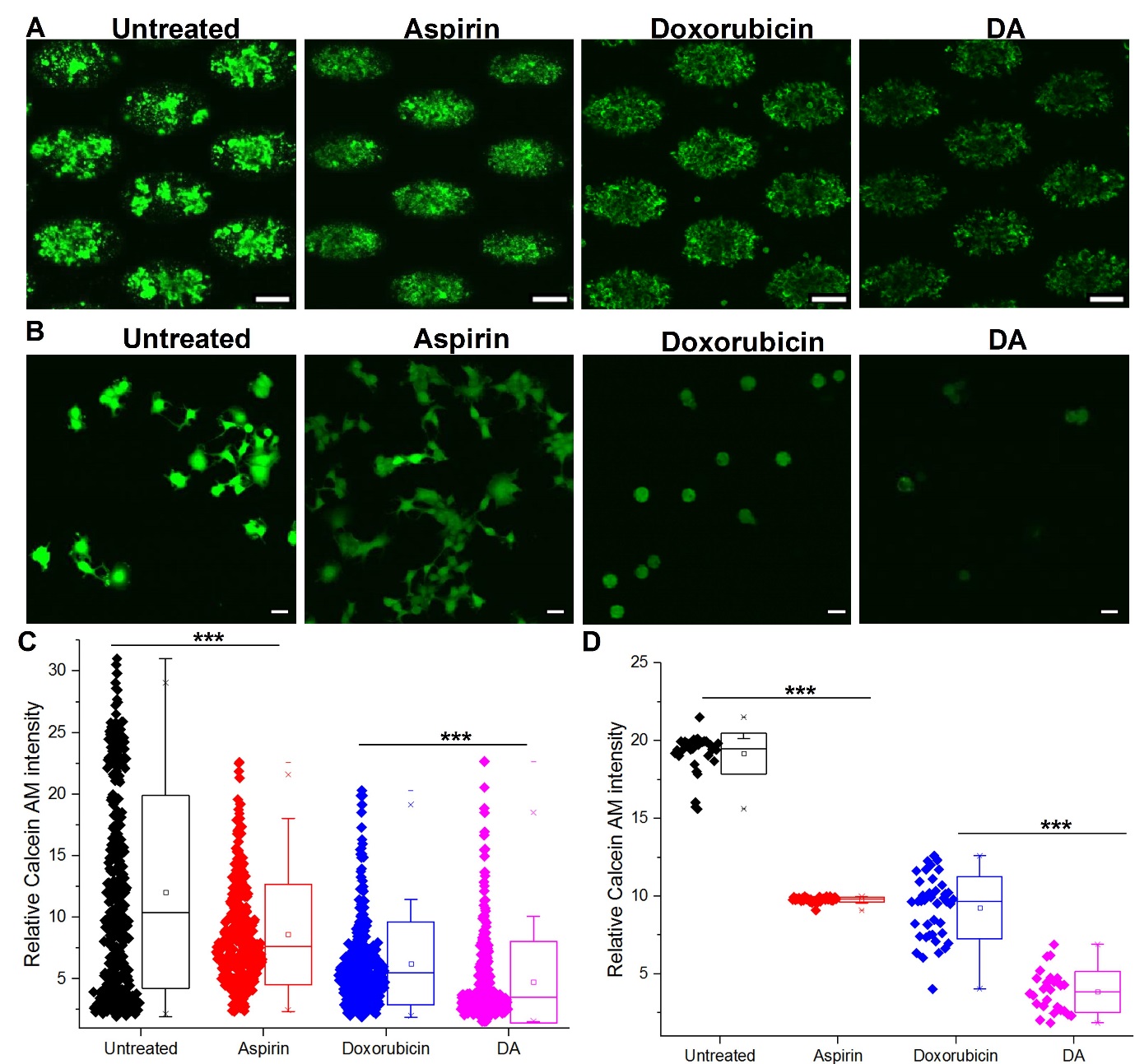


**Fig. S15 Staining intensity of Calcein AM under different conditions.** (A) Staining of Calcein AM of cancer cell clusters *in situ*. Scale bar is 100 µm. (B) Staining of Calcein AM of harvested cells. Scale bar is 20 µm. (C) Comparison of relative fluorescence intensity of Calcein AM relative to respective background values (Cluster cultures). *** *p* < 0.00001. (D) Comparison of relative fluorescence intensity of Calcein AM relative to respective background values (2D cultures). *** *p* < 0.00001.


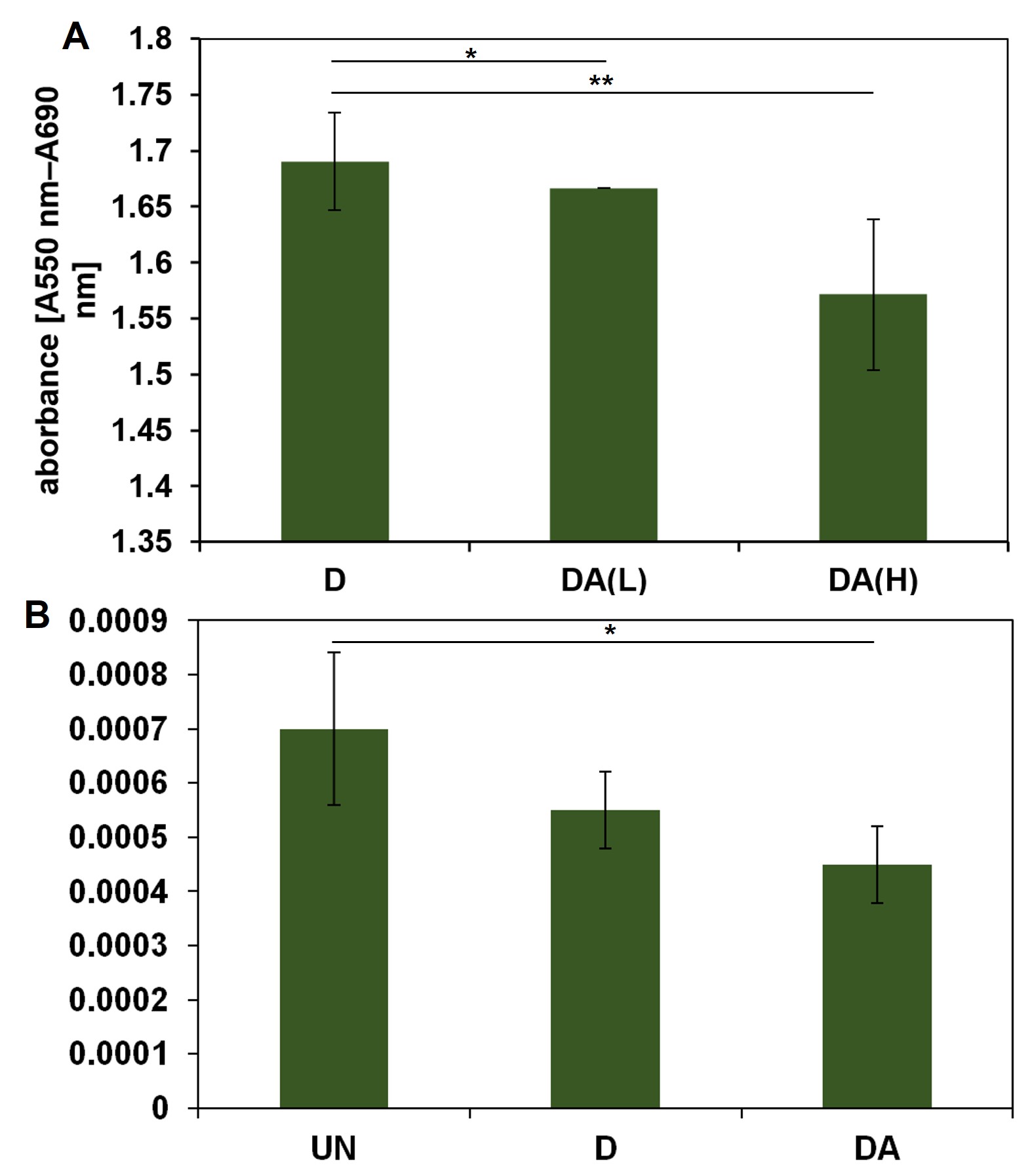


**Fig. S16 Assays for evaluation of the relative metabolic activity.** (A) The difference in absorbance values under MTT assay after 72 h treatment with single doxorubicin treatment (D), or combination treatment with low (DA(L)) or high aspirin (DA(H) concentrations. Values are normalized to untreated control. (B) Relative absorbance values under peroxidase assay after 72 h treatment with untreated control, single doxorubicin treatment (D), or combination treatment (DA). * *p* < 0.01, ** *p* < 0.001.

**
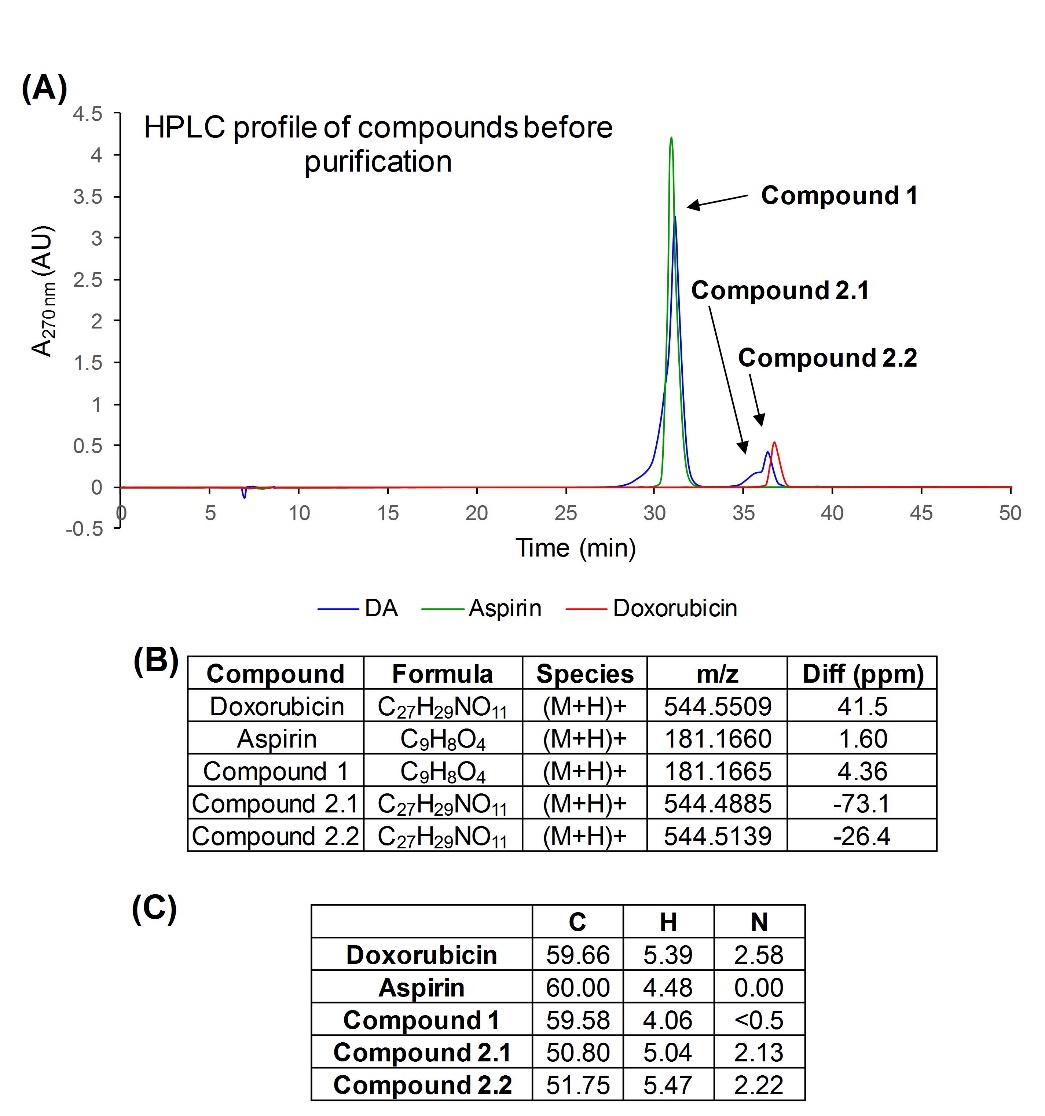
**

**Fig. S17** Purification and analysis of compounds formed under DA combination. (A and B) HPLC and MS analysis of various purified compounds. Compound 1 in the combinatorial DA mix was likely to be aspirin while compounds 2.1 and 2.2 seem to reflect doxorubicin and were likely to be impurities. (C) CHNS elemental analysis of various compounds.


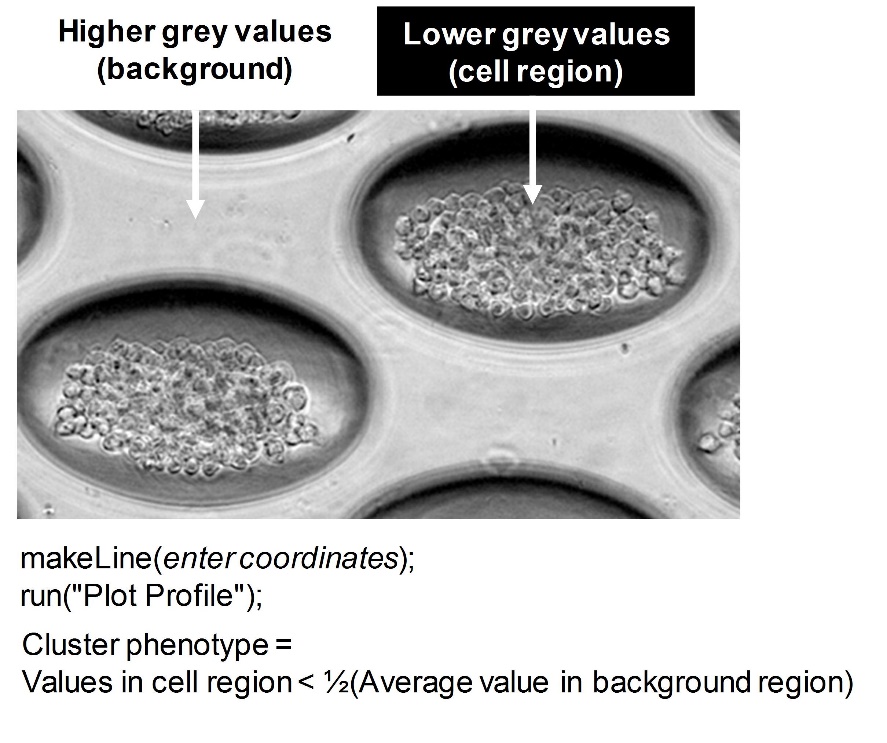


**Fig. S18. Determining the presence of cluster phenotype.** Grey values in two regions: 1) Background (non-microwell) and 2) Cell region (within microwell) were obtained with image processing software. In cancer cell clusters, greyscale values within the cell region will be lower than half of the average grey scale value computed from the background region.

**Supplementary Tables**

**Table S1 Clinical samples and patient demographics.** CES, BC, and ASL stand for cohort types. NA = not available. ER = estrogen, PR = progesterone, HER2 = human epidermal growth factor receptor 2. ‘1’ denotes positive result, ‘0’ denotes negative result. Treatment types include but are not limited to Docetaxel, Doxorubicin, PMRT, Lapatinib, Tamoxifen, Herceptin, Exemestane, Fulvestrant, ASLAN/Taxol, Letrazole, RT.

| **ID** | **Time point** | **Cancer Stage** | **Age** | **Tumor /mm** | **Hormone Receptor Status** | | | **Cluster forming potential** | | |
| --- | --- | --- | --- | --- | --- | --- | --- | --- | --- | --- |
|  |  |  |  |  | **ER** | **PR** | **HER2** | **< 30%** | **30-60%** | **> 60%** |
| ASL32 | EOT | IIIB | 49 | NA | 1 | 0 | 1 | 1 |  |  |
| ASL36 | Post treatment | IIA | 39 | NA | 0 | 0 | 1 |  |  | 1 |
| BC234 | Post-treatment | IIB | 53 | 29 x 21 x 37 and 20 x 17 x 19 mm | 1 | 0 | 1 |  |  | 1 |
| BC239 | Pre-treatment | IV | 54 | 42 x 40 x 22 | 1 | 1 | 0 |  | 1 |  |
| BC240 | Pre-treatment | IV | 48 | 54 x 70 x 61 | 1 | 0 | 0 |  | 1 |  |
| BC241 | Pre-treatment | IV | 59 | 112 x 93 | 1 | 0 | 0 |  | 1 |  |
| BC247 | Pre-treatment | I | 70 | 15 | 1 | 1 | 0 |  |  | 1 |
| BC249 | Pre-treatment | IV | 63 | 25 x 14 x 30 | 1 | 0 | 1 |  |  | 1 |
| CES093 | Post treatment | IIB | 60 | NA | 0 | 0 | 1 | 1 |  |  |
| CES093 | Post treatment | IIB | 60 | NA | 0 | 0 | 1 |  |  | 1 |
| CES102 | Post treatment | IIIA | 67 | 50 | 1 | 1 | 0 |  |  | 1 |
| CES105 | Post treatment | IA | 69 | 17 | 0 | 0 | 1 |  |  | 1 |
| CES16 | Post treatment | IIA | 61 | 14 | 0 | 0 | 0 |  | 1 |  |
| CES64 | Post treatment | 0 | 50 | 10 | 1 | 1 |  |  |  | 1 |
| CES76 | Post-treatment | I | 66 | 13 | 1 | 0 | 0 |  |  | 1 |
| CES78 | Post treatment | I | 66 | 9 and 3 | 1 | 1 | 0 |  |  | 1 |
| CES83 | Post-treatment | IA | 53 | 2 | 1 | 1 | 0 |  |  | 1 |

**Table S2 Aspirin intake period and dosage from various clinical cohorts.** ND: Not determined.

| **Cancer type** | **Daily dosage/ mg** | **Duration/ years** | **Observations** | **Latency/ years** | **Ref** |
| --- | --- | --- | --- | --- | --- |
| Colorectal | 500 | 20 | Reduced cancer incidence | 10 | (*61*) |
|  | 81 | 5 | 19% reduced risk | ND | (*62*) |
|  | 325 | 5 | No reduced risk | ND |  |
|  | 325 | 5 | Chemopreventive | ND | (*63*) |
|  | 75-300 | 20 | Reduced incidence of cancer |  | (*8*) |
| Rectal | 75-300 | 20 | Did not reduce risk |  |  |
| Esophageal | ND | 12 to 16 | 90% decreased risk | 11 | (*64*) |
| Gastric | ND | 6 | Decreased risk; dose-dependent | 5 | (*9*) |
| Breast | ND | 1 | Decreased risk of distant recurrence and death | ND | ^(^*^65^*^)^ |

**Table S3 Sample list for CTC Cluster Assay.** CES, BC, and ASL stand for cohort types. Clinical timepoints: EOT = end of treatment, PD = Post drug, ‘Y’ denotes positive result, ‘N’ denotes negative result. Treatment types include but are not limited to Docetaxel, Doxorubicin, PMRT, Lapatinib, Tamoxifen, Herceptin, Exemestane, Fulvestrant, ASLAN/Taxol, Letrazole, RT.

|  | **ID** | **Timepoint** | | **Cluster Positivity** |
| --- | --- | --- | --- | --- |
| 1 | ASL010 | 24 weeks | PD | Y |
| 2 | ASL010 | EOT | PD | N |
| 3 | ASL011 | 16 weeks | PD | N |
| 4 | ASL012 | 12 weeks | PD | N |
| 5 | ASL012 | 14 weeks | PD | N |
| 6 | ASL012 | 16 weeks | PD | Y |
| 7 | ASL012 | 20 weeks | PD | Y |
| 8 | ASL015 | 16 weeks | PD | N |
| 9 | ASL015 | 18 weeks | PD | Y |
| 10 | ASL015 | 12 weeks | PD | N |
| 11 | ASL024 | 22 weeks | PD | N |
| 12 | ASL029 | 4 weeks | PD | N |
| 13 | ASL029 | 6 weeks | PD | N |
| 14 | ASL029 | 8 weeks | PD | N |
| 15 | ASL032 | Pre-treatment | B | N |
| 16 | ASL032 | 4 weeks | PD | N |
| 17 | ASL032 | 6 weeks | PD | N |
| 18 | ASL032 | EOT | PD | Y |
| 19 | ASL035 | 4 weeks | PD | N |
| 20 | ASL035 | EOT | PD | N |
| 21 | ASL036 | 4 weeks | PD | N |
| 22 | ASL036 | 6 weeks | PD | Y |
| 23 | ASL044 | BASELINE | B | Y |
| 24 | ASL044 | C3D1 | PD | Y |
| 25 | ASL045 | C2D1 | PD | Y |
| 26 | BC130 | Pre-treatment | B | Y |
| 27 | BC215 | Pre-treatment | B | Y |
| 28 | BC233 | 4 weeks | PD | Y |
| 29 | BC233 | Pre-treatment | B | Y |
| 30 | BC234 | Pre-treatment | B | Y |
| 31 | BC234 | Pre-treatment | B | Y |
| 32 | BC236 | Pre-treatment | B | N |
| 33 | BC236 | Pre-treatment | B | Y |
| 34 | BC237 | Pre-treatment | B | N |
| 35 | BC239 | Pre-treatment | B | Y |
| 36 | BC239 | 4 weeks | PD | Y |
| 37 | BC240 | Pre-treatment | B | Y |
| 38 | BC241 | Pre-treatment | B | Y |
| 39 | BC245 | Pre-treatment | B | Y |
| 40 | BC245 | 4 weeks | PD | Y |
| 41 | BC249 | Pre-treatment | B | Y |
| 42 | BC249 | 4 weeks | PD | Y |
| 43 | BC251 | Pre-treatment | B | Y |
| 44 | BC261 | Pre-treatment | B | Y |
| 45 | BC262 | Pre-treatment | B | N |
| 46 | BC263 | Pre-treatment | B | Y |
| 47 | BC264 | Pre-treatment | B | N |
| 48 | BC265 | Pre-treatment | B | Y |
| 49 | BC266 | Pre-treatment | B | Y |
| 50 | BC267 | Pre-treatment | B | N |
| 51 | CES016 | 3 years | PD | Y |
| 52 | CES040 | 1 year | PD | N |
| 53 | CES093 | 1 year | PD | Y |
| 54 | CES102 | > 1 year | PD | Y |
| 55 | CES102 | Post-Surgery | PD | Y |
| 56 | CES104 | > 1 year | PD | N |
| 57 | CES104 | Post-Surgery | PD | Y |
| 58 | CES105 | 1 year | PD | N |
| 59 | CES105 | > 1 year | PD | Y |
| 60 | CES106 | > 1 year | PD | Y |
| 61 | CES107 | > 1 year | PD | Y |
| 62 | CES107 | > 1 year | PD | N |
| 63 | CES108 | Pre-treatment | B | N |
| 64 | CES129 | 1 year | PD | Y |
| 65 | CES173 | 2ND; POST NEOADJ TAXOL | PD | Y |
| 66 | DDAC012 | Post-Surgery | PD | N |
| 67 | DDAC043 | 6 weeks | PD | Y |
| 68 | LL-005 | AFT 4WK DA | PD | Y |

**Table S4. List of all gene primers used in this study.**

| **mRNA** | **F/R** | **Primers from 5' to 3'** |
| --- | --- | --- |
| JAK1 | Forward | CTTTGCCCTGTATGACGAGAAC |
| JAK1 | Reverse | ACCTCATCCGGTAGTGGAGC |
| JAK2 | Forward | TCTGGGGAGTATGTTGCAGAA |
| JAK2 | Reverse | AGACATGGTTGGGTGGATACC |
| JAK3 | Forward | TTCGGGCTACGCAAGGATTTG |
| JAK3 | Reverse | AGGCTGAGACACTCACCCT |
| STAT1 | Forward | CAGCTTGACTCAAAATTCCTGGA |
| STAT1 | Reverse | TGAAGATTACGCTTGCTTTTCCT |
| STAT2 | Forward | GAGCCAGCAACATGAGATTGA |
| STAT2 | Reverse | GCCTGGATCTTATATCGGAAGCA |
| STAT3 | Forward | CAGCAGCTTGACACACGGTA |
| STAT3 | Reverse | AAACACCAAAGTGGCATGTGA |
| STAT5 | Forward | TGCTGGCCGAGGTCAAC |
| STAT5 | Reverse | AGACTTGGCCTGCTGCTCAC |
| GAPDH | Forward | CAAGCTCATTTCCTGGTATGAC |
| GAPDH | Reverse | CAGTGAGGGTCTCTCTCTTCCT |
| UBB | Forward | GCTTTGTTGGGTGAGCTTGT |
| UBB | Reverse | CGAAGATCTGCATTTTGACCT |
